# Repurposing of H_1_-receptor antagonists (levo)cetirizine, (des)loratadine, and fexofenadine as a case study for systematic analysis of trials on clinicaltrials.gov using semi-automated processes with custom-coded software

**DOI:** 10.1007/s00210-023-02796-9

**Published:** 2023-10-23

**Authors:** Tim Specht, Roland Seifert

**Affiliations:** Hannover Medical School, Institute of Pharmacology, Carl-Neuberg-Str. 1, 30625 Hannover, Germany

**Keywords:** Drug repurposing, H_1_-receptor antagonists, Information storage and retrieval, Clinical trials as topic

## Abstract

To gain a comprehensive overview of the landscape of clinical trials for the H_1_-receptor antagonists (H_1_R antagonists) cetirizine, levocetirizine, loratadine, desloratadine, and fexofenadine and their potential use cases in drug repurposing (the use of well-known drugs outside the scope of the original medical indication), we analyzed trials from clincialtrials.gov using novel custom-coded software, which itself is also a key emphasis of this paper. To automate data acquisition from clincialtrials.gov via its API, data processing, and storage, we created custom software by leveraging a variety of open-source tools. Data were stored in a relational database and annotated facilitating a specially adapted web application. Through the data analysis, we identified use cases for repurposing and reviewed backgrounds and results in the scientific literature. Even though we found very few trials with published results for repurpose indications, extended literature research revealed some prominent use cases: Cetirizine seems promising in mitigating infusion-associated reactions and is also more effective than placebo in the treatment of androgenetic alopecia. Loratadine may be beneficial in the prophylaxis of G-CSF-related bone pain. In COVID-19, H_1_R antagonists may be helpful, but placebo-controlled scientific evidence is needed. For asthma, the effect of H_1_R antagonists only seems to be secondary by alleviating allergy symptoms. Our novel method to find potential use cases for repurposing of H_1_R antagonists allows for high automation, reduces human error, and was successful in revealing potential areas of interest. The software could be used for similar research questions and analyses in the future.

**Supplementary Information:**

The online version contains supplementary material available at 10.1007/s00210-023-02796-9.

## Introduction

H_1_-receptor antagonists, such as cetirizine, loratadine, and fexofenadine, are widely used in the treatment of a variety of allergic conditions, including rhinitis, conjunctivitis, hay fever, and urticaria. These drugs are well-tolerated, extensively tested, and cost-effective. Therefore, H_1_-receptor (H_1_R) antagonists are promising candidates for drug repurposing.

As suggested by the name, H_1_R antagonists act against the H_1_ receptor, one of four important G-protein coupled receptors in the histaminergic system. Histamine itself is a biogenic amine that functions as a neurotransmitter and local mediator through the already mentioned H_1–4_ receptors. Being a biogenic amine, histamine is derived from histidine and is mostly stored in mast cells, basophils, and enterochromaffin-like (ECL) cells of the gastrointestinal tract (Seifert 2019, p. 94).

The four histamine receptors can be found at different locations, and they are responsible for various actions. For H_1_R antagonists, of course, the H_1_ receptor is of great importance and is involved in allergic reactions like pruritus, erythema, and edema. Also, behavioral effects and modulation of immune cells could be observed (Borriello et al. 2017).

Effects on immune cells were also associated with the H_2_ receptor, which also induces hydrochloric acid secretion in gastric parietal cells. The H_3_ receptor is mainly involved in the central nervous system (CNS) for the regulation of behavior and body temperature and reduction of norepinephrine release. Lastly, the H_4_ receptor again plays a role in the regulation of immune cells and is involved in allergic and immunologic disorders like asthma (Borriello et al. 2017).

Cetirizine, levocetirizine, loratadine, desloratadine, and fexofenadine are antihistamines, more precisely, H_1_R antagonists, and therefore inhibit the H_1_ receptor and the effects its activation has. They are primarily and classically used to treat allergic conditions, including rhinitis, conjunctivitis, hay fever, and urticaria (Seifert 2019, p. 96).

H_1_R antagonists can be divided into the first and second generation. The first generation of H_1_R antagonists (e.g., diphenhydramine or dimetindene) is relatively unspecific and, most notably, can pass through the blood–brain barrier due to their more lipophilic nature. Here, they cause drowsiness and other CNS adverse effects (Seifert 2019, p. 96).

The newer second-generation antagonists can also pass through the barrier to the CNS; there are, however, transporters that transport these newer compounds back into the blood away from the CNS and therefore limit the adverse CNS effects when used within reasonable dosages.

Widely known, cetirizine is a popular over-the-counter drug to treat allergy symptoms. Levocetirizine is the R-enantiomer of the racemic compound cetirizine. Similarly, loratadine is an alternative to cetirizine, while desloratadine is the active metabolite of loratadine. Fexofenadine is the active metabolite of terfenadine. Terfenadine itself is no longer in use because of potential cardiotoxicity (Panula et al. 2015).

Being well-studied, inexpensive, and widely available, these second-generation H_1_R antagonists are promising candidates for drug repurposing. “Drug repurposing (also called drug repositioning, reprofiling, or re‑tasking) is a strategy for identifying new uses for approved or investigational drugs that are outside the scope of the original medical indication” (Pushpakom et al. 2019). The advantages are apparent: the potentially repurposed drugs have already been sufficiently tested and found to be safe in preclinical models and humans. Therefore, the timeframe for development and the associated costs are significantly reduced. A prominent example for a repurposed drug is sildenafil. While originally used in angina pectoris, it was then developed to be used in treatment of erectile dysfunction through “retrospective clinical experience” (Pushpakom et al. 2019).

To gain a comprehensive overview of the state of repurposing of the H_1_R antagonists cetirizine, levocetirizine, loratadine, desloratadine, and fexofenadine, we turned to clinicaltrials.gov as a starting point to answer the question which indications are being studied in the context of repurposing of H_1_R antagonists and what these findings could entail for the future of these drugs. Additionally, with regard to our method, we wanted to evaluate and test our novel approach of systematical and semi-automated processing and analysis of data from clinicaltrials.gov using custom-coded software to see if it is viable and maybe even a transferable approach for similar analyses.

## Methods

Clinicaltrials.gov is the US National Library of Medicine’s publicly available database for clinical trials and studies that went online in 2000 and, as of mid-2023, lists over 450 thousand trials. However, it is not only limited to studies and trials conducted in the USA but also lists studies in over 200 countries. Sponsors and investigators are responsible for submitting and updating information on their respective trials, which can then be found by patients and researchers alike (https://clinicaltrials.gov/search, last accessed July 3, 2023; https://clinicaltrials.gov/about-site/about-ctg, last accessed July 3, 2023).

For systematic and convenient access to the data stored by clinicaltrials.gov, an API is provided in addition to various export options through the front-end website (the part of the website that is visible to every user and can be navigated with a keyboard and mouse visually). API is the abbreviation for “application programming interface” (https://classic.clinicaltrials.gov/api/gui, last accessed July 3, 2023).

Those interfaces are common for websites and databases and provide data access based on structured search terms and queries. In the case of the clinicaltrials.gov API, results are then formatted automatically in XML or JSON, which are very specific notations so that they can easily be processed by computer programs. We used the JSON (= JavaScript Object Notation) format because it fits our technologies used more efficiently. However, it needs to be noted that the data provided by both formats is identical, and a conversion is possible.

**Fig. 1:**
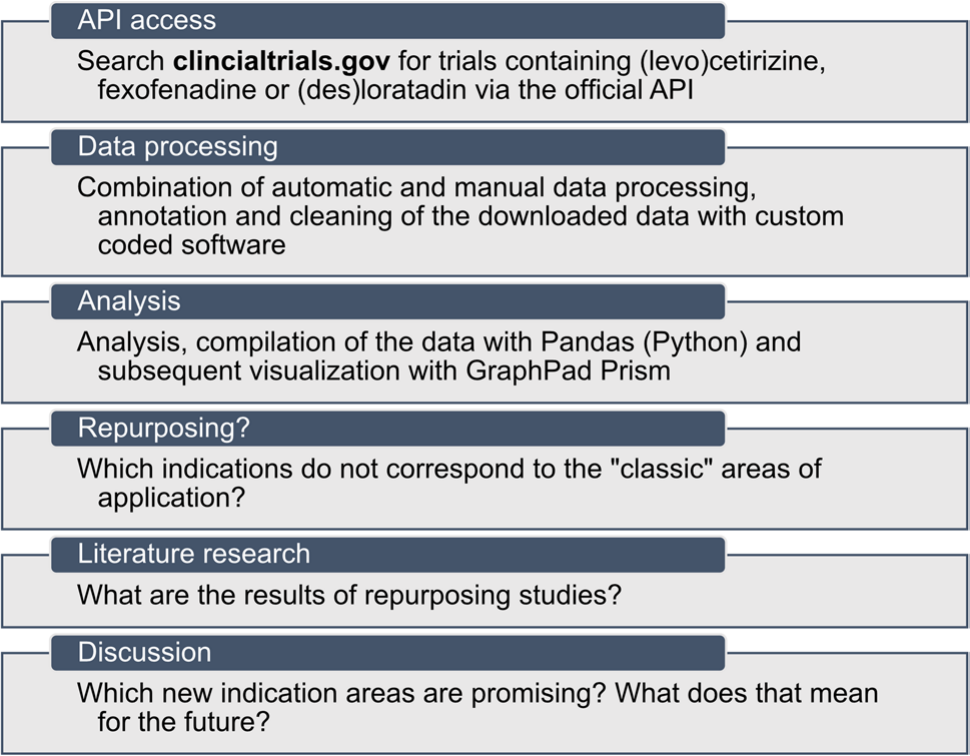
This flowchart shows a high-level abstracted overview of our method

Figure 1 shows an overview of our method. The following will explain the steps listed in detail, as well as an overview from the technical side.

As a first step, we downloaded study data via the API mentioned above (as of January 2023). Here, the API endpoint “https://clinicaltrials.gov/api/query/study_fields” was used. This specific endpoint “returns values from selected API fields for a large set of study records” (https://classic.clinicaltrials.gov/api/gui/ref/api_urls, last accessed July 3, 2023). Relevant fields were identified and selected for download, for example:

- NCTId (unique identifier for each study assigned by clinicaltrials.gov)
- Basic information about the study, e.g., “OfficialTitle,” “BriefTitle,” “BriefSummary,” “Conditions,” “Gender,” “MaximumAge,” “MinimumAge”
- Information about study progress and other metadata: “OverallStatus,” “StartDate,” “CompletionDate,” “LastUpdatePostDate,” etc.

A full list of the downloaded fields can be found in the provided source code in the file “data/api_download.ts”. As the search expression, “Cetirizine OR Levocetirizine OR Fexofenadine OR Loratadine OR Desloratadine” was used, which also includes synonyms or other drug names automatically (e.g., Zyrtec® for cetirizine).

As mentioned, APIs are used to access data via program code. Therefore, we leveraged custom-coded software to download and process the study data. Because of the compatibility with web technologies, which will become important for the next step of manual data annotation, TypeScript was chosen as the primary programming language.

TypeScript is a language created initially by Microsoft that builds on top of and extends JavaScript, the programming language which mostly powers websites on the internet. As the name suggests, TypeScript extends JavaScript with types. To explain in simple terms, a variable is a “container” to store data. In JavaScript, a variable can store anything, regardless if it is a text (so-called strings), a number, or a complex object. While this seems like an advantage at first, it soon can become too complex and too flexible and is prone to errors. TypeScript makes it possible to give these containers specific types and therefore limits the data that can be assigned (for example, only numbers can be stored in a given variable) and thus eliminates errors that were previously easily made in JavaScript (https://www.typescriptlang.org/why-create-typescript, last accessed July 3, 2023). NodeJS was used to run TypeScript locally on a computer outside the context of a web browser (https://nodejs.org/, last accessed July 3, 2023).

For the download, the script “data/api_download.ts” was created, which essentially downloads study data through the API with the selected fields and for the search expression mentioned above and then stores the data locally in a file. Because of API limitation, the maximum number of studies that can be downloaded at once is 1000. This, however, is sufficient for the given search expression because there are only about 400–500 studies that match the expression of the H_1_R antagonists (this can be checked beforehand by using the normal website of clinicaltrials.gov with the same search expression).

After downloading, the data needed to be processed, annotated, and verified before analysis. First, we processed the data automatically wherever reasonable, again using a TypeScript script (“data/processing.ts”).

First and foremost, the previously downloaded data was read from the temporary file storage. These data are then checked for missing values and whether the format and result provided by the API are as expected for further processing.

The API returns some fields as an array (a list of values), even though there is only one element in the list (like the NCTId). These arrays are flattened and replaced by the single value.

For these fields, some values need to be processed further, like the ages and dates, because they have inconsistent formatting by default. Some ages are provided in months, most in years; the dates sometimes miss the day and only include month and year. For these cases, transformer functions were created which detect these irregularities and return data with uniform formatting.

As an example, the “ageTransformer” (data/processing.ts, ll. 60ff) will be explained in detail to provide a high-level overview.

**Fig. 2:**
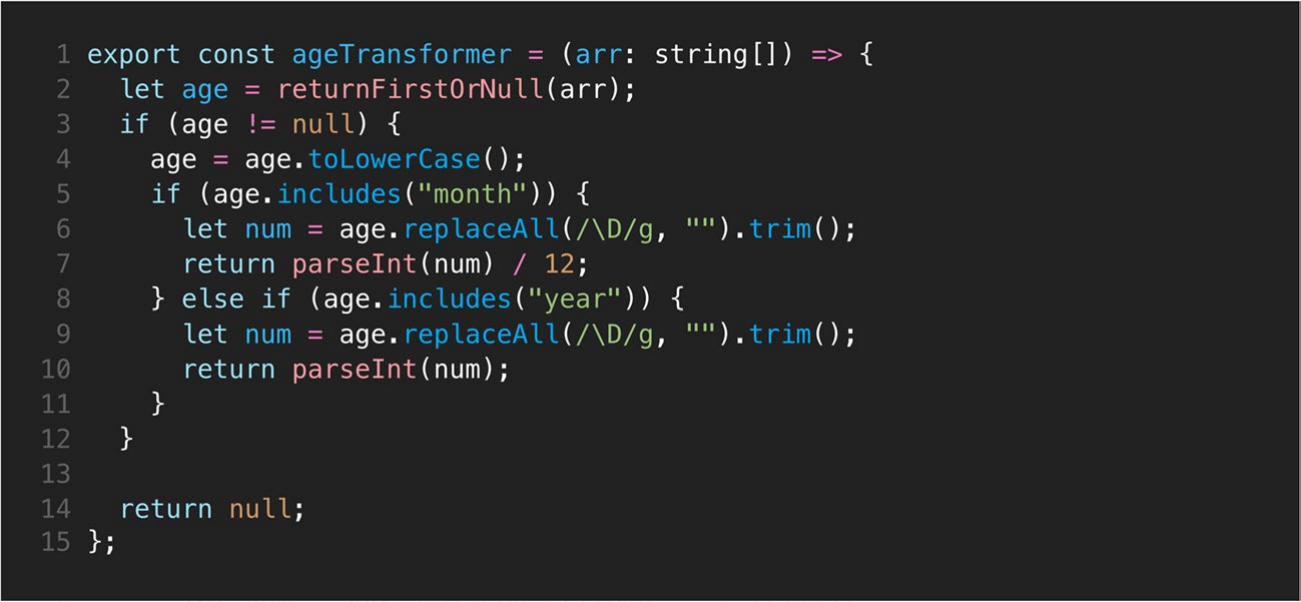
The “ageTransformer” function formats a given array of strings returned by the clinicaltrials.gov API as a uniform date string so it can be used later in the analysis

Figure 2 shows the code of the ageTransformer functions that expects a list of text as an input (“arr: string[],” line 1). This is because the API returns the age as a list of text, for example (“11 Years”) or (“6 Months”). On the second line, the first list item is extracted. If it exists (otherwise, it would be “null”), it is transformed to lowercase letters (l. 4). It is checked if the age is provided in months (l. 5) or in years (l. 8). Regardless of the unit, the number is extracted via a so-called regular expression (l. 6, all characters which are not a number are being deleted or rather replaced by an empty string). If the age was provided in months, the result is divided by 12 to get the age in years (l. 7).

The dateTransformer function works similarly, formatting the data uniformly by setting the day to the first of the month if it is not provided and returning the date as a string of “YYYY-MM-DD,” e.g., “2000–08-06” for the 6th of August 2000.

After processing single values, data fields containing a list of values, like conditions (MeSH terms) or locations, are scanned for unique values. This aims to eliminate duplicate data in the next step when transferring the data for permanent storage to a database. After finding the unique values, each value is assigned a unique id (just a number increased by 1 for each new term).

As a last step, the processed data are stored in a PostgreSQL database (https://www.postgresql.org/, last accessed July 3, 2023) through a translations layer called Prisma (https://www.prisma.io, last accessed July 3, 2023) which is an ORM specifically developed for the use with TypeScript. ORM is the abbreviation for “object-relational mapping,” which is necessary when transferring data from TypeScript, which works with objects, to a relational database, like PostgreSQL, which works more like a collection of connected Excel tables. Here, data are stored in rows and columns, and relations between data entries are established via ids and references, which is another reason why the unique values of list fields had to be extracted and assigned individual ids.

By leveraging SQL queries, the study data can be filtered, connections (so-called unions) can be made, or database entries can be modified.

**Fig. 3:**
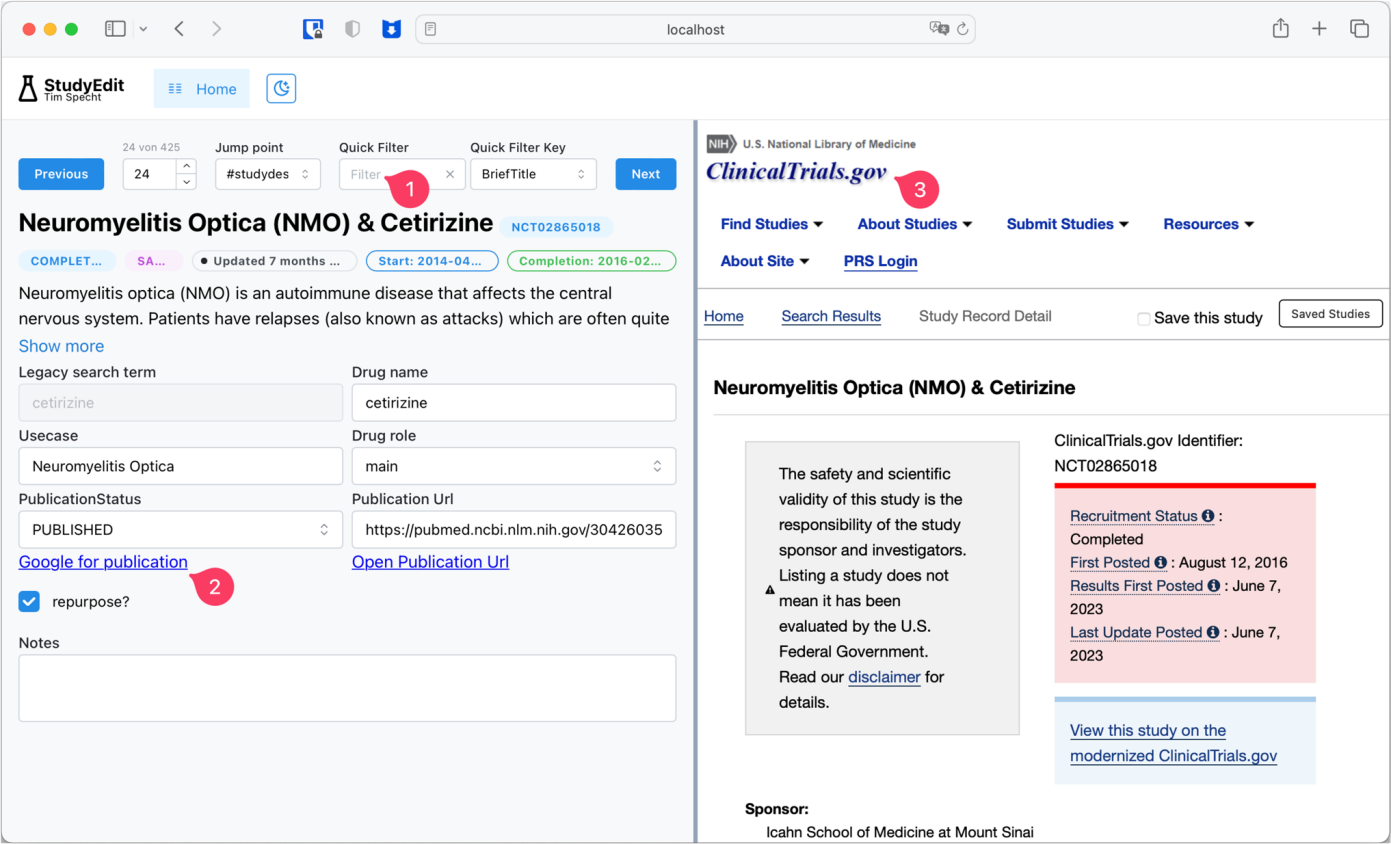
Web application for manual annotation with quick filter (1), link shortcuts (2), and simultaneous view of the study’s webpage (3). It can be accessed via a regular web browser (here, Safari on macOS) but is only available in the local network and not via the internet

Manual annotation and verification are necessary after these steps of automatic data processing and storage. To make this step as seamless and easy as possible, a custom web application was created (we called “StudyEdit,” Fig. 3) that uses the same technologies already described but with bidirectional dataflow.

The now populated PostgreSQL database is again connected through Prisma ORM to a local API run with TypeScript, which can be accessed through a web frontend that makes it easy to view the clinicaltrials.gov website for the study on the right-hand side and to be able to edit and annotate the data on the left-hand side. The data editor is custom-tailored to the data points and annotations of interest. Also, “quality of life shortcuts” are integrated, like a button to search for study results on the internet, should they not have been provided by the study authors. The front-end was built with NextJS, which uses ReactJS, and tRPC is used for connectivity.

For manual data annotation, each study was assigned additional fields. A value for the field “drug name” was added manually based on the H_1_R antagonist(s) used in the trial. Because the API results contained all studies for every H_1_R antagonist of interest, an automatic association was not possible. Furthermore, even earlier methods of data downloading did not result in reliable assignment of drug names (represented by the field legacy search term; here, we downloaded data individually per H_1_R antagonist on our list). Wherever there were multiple H_1_R antagonists included in the respective study, the “drug name” was chosen by individual consideration and, if no clear preference could be found, by the order they appeared in the study. However, these additional H_1_R antagonists were also noted in the field, but grouping and categorization in the analysis were done with regard to the first drug name.

A use case was also assigned manually based on the role of the H_1_R antagonist in the study. This use case did not necessarily match the primary condition of the study. For example, in a study about multiple sclerosis, an H_1_R antagonist might have only been used in the treatment of infusion-associated reaction, which was consecutively assigned as the use case and *not* multiple sclerosis.

Additionally, the role of the H_1_R antagonist in the study was matched into one of the following categories:

- Combination: H_1_R antagonist is combined with some other form of treatment.
- Comparison: H_1_R antagonist is compared to some other drugs or forms of treatment.
- Control: H_1_R antagonist is a control drug and not an aspect of focus in the study.
- Main: H_1_R antagonist is the main focus of the study.
- Minimal: H_1_R antagonist has a very minimal role in the study.
- Not included: H_1_R antagonists are not included in the study (sometimes names of people also matched synonyms of search terms).
- Premedication: H_1_R antagonist is used as a premedication and not evaluated further.
- Rescue medication: H_1_R antagonist is used as a rescue medication.

Furthermore, a checkmark in the web interface could be selected if the study fell into the general direction of repurposing. In a second step, where not provided by study authors, we tried to find publications of study results through PubMed and Google for those studies marked as repurposing. All these annotations were directly written to the database again, so it provided the only and single source of truth for the data.

After manual annotation, an analysis was conducted of the general study data using Python, another programming language. Here, the Pandas library (https://pandas.pydata.org/, last accessed July 22, 2023) was leveraged, which is built specially for data analysis.

Data were imported from the PostgreSQL database into memory in a pandas dataframe, and pivot tables were created using the built-in functions “pivot_table” and “crosstab.” These transformed data were then saved as a CSV file temporarily for import into GraphPad Prism for visualization. Alternatively, a (preview) visualization is also available in the corresponding Jupyter notebook using Matplotlib.

Lastly, it is most notable that all this processing, apart from visualization with GraphPad Prism, could be performed for free using open-source tools and software. A summary of the most important open-source tools we used can be seen in Table 1. In addition to those tools listed, we also used smaller tools and libraries with specific tasks like HTTP requests (axios) or plotting data (Matplotlib) separately from GraphPad Prism.

**Table 1:** Selection of the important open-source programming languages, tools, and software used as building blocks to create our custom software

Name	Link	General use case	What we used it for
TypeScript	https://www.typescriptlang.org/	Programming language (extended version of JavaScript introduced by Microsoft)	Scripts for data download and processing, building web application
NodeJS	https://nodejs.org/	JavaScript runtime to use it outside the context of a web browser	Running TypeScript scripts
PostgreSQL	https://www.postgresql.org/	Relational database for data storage	Storing data after download and processing
Prisma	https://www.prisma.io/	ORM system (object relational mapping) for TypeScript for easy access to data stored in a relational database	Writing data to the database after downloading and processing, reading, and writing data through the web application
ReactJS	https://react.dev/	Library to create user interfaces for the web	Web application user interface
NextJS (T3 Stack)	https://nextjs.org/ https://create.t3.gg/	Frameworks that incorporate ReactJS for the creation of full stack (= front and back end) web applications	Web application for study annotation and viewing
Python	https://www.python.org/	Programming language	Data analysis, export to CSV
Pandas	https://pandas.pydata.org/	Data analysis library for Python	Data analysis, especially pivot tables

Our source code is available on GitHub at https://github.com/T-Specht/h1ra-repurpose/.

Additionally, an instructional video for the basic installation and usage of the software and its components can be accessed at the link above.

## Results and discussion

### Dataset analysis

To gain more insight into the general state of research for the H_1_R antagonists of interest, we first conducted a general analysis of the metadata of all studies we found.

**Fig. 4:**
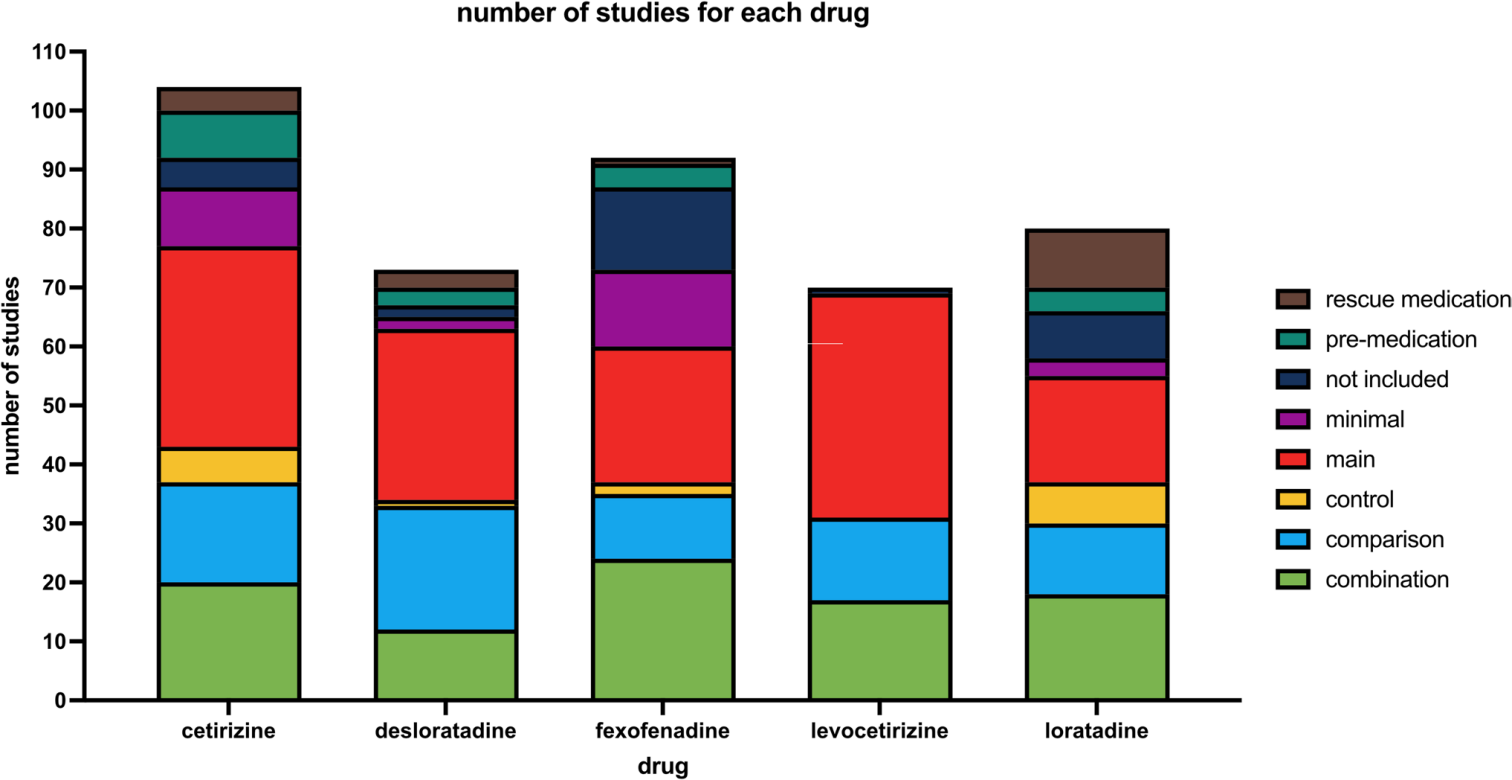
The absolute number of studies grouped by drug name with color-coded drug roles, which were assigned manually. This is the only chart in which all drug roles, including “minimal,” “not included,” or “rescue medication” are visualized. In every other chart, the mentioned categories were excluded

Figure 4 shows the number of studies grouped by the drug name. For each drug, there are between 70 (levocetirizine) and 104 (cetirizine) studies that matched our search expression, the most for cetirizine. As already mentioned, it was not always possible to assign only one drug to the study; therefore, only the first drug name listed is considered in Fig. 4. Apart from the categories “main,” “comparison,” and “combination,” there were quite a few studies in which the H_1_R antagonists of interest were not included at all. This was most notably the case with fexofenadine, where this phenomenon could be the case because of the trade name “allegra” which came up in the study as the name of some person involved.

To limit the following figures to relevant studies only, studies in which the previously described drug role was assigned as “minimal,” “not included,” or “rescue medication” were *excluded*.

**Fig. 5:**
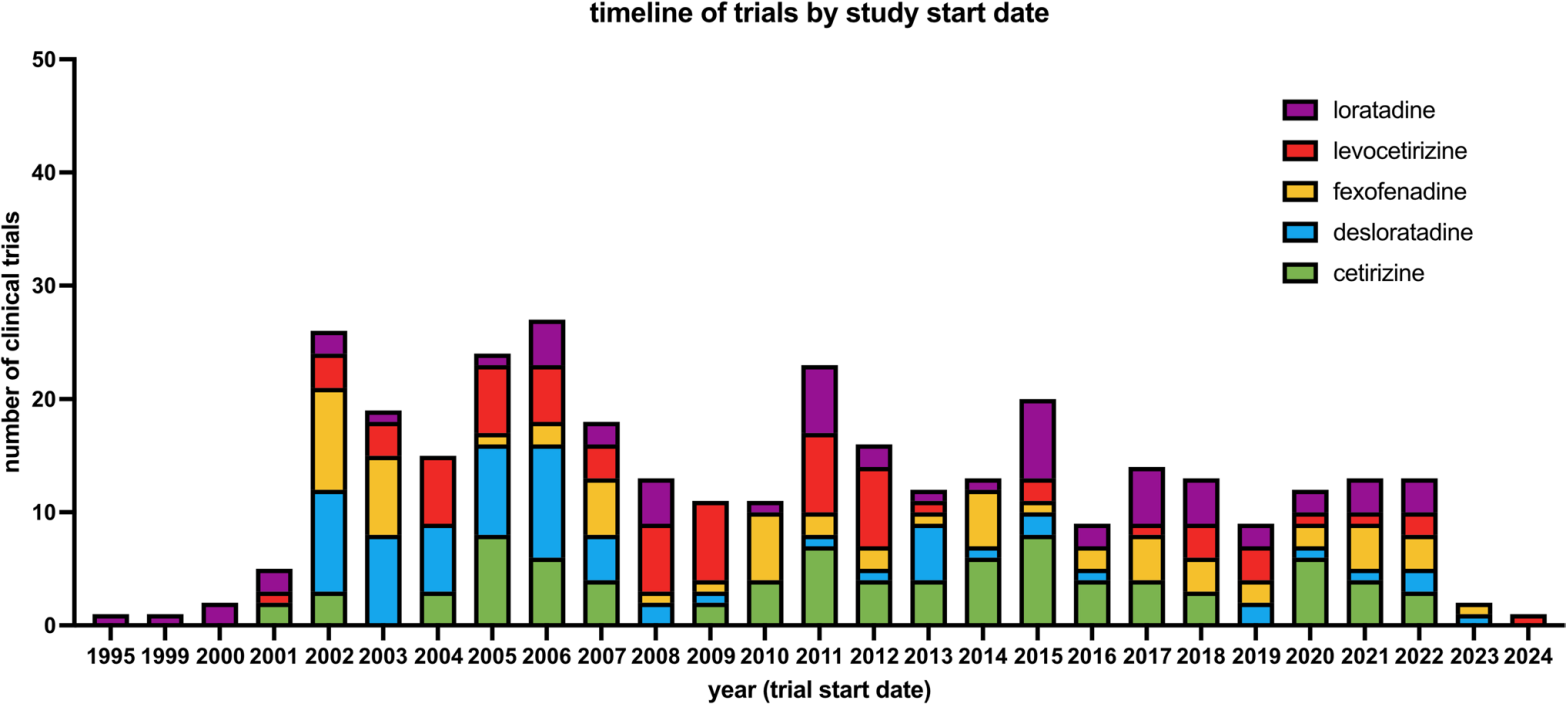
This chart shows the absolute number of studies grouped by year based on the study start date field, color-coded by drug name. Also, refer Supplement Fig. 1 for the same graph, but based on the date, the study was first posted on clinicaltrials.gov

Figure 5 shows the timeline of studies by study start date. Unsurprisingly, there are very few studies that started before 2000, as clinical-trials.gov went online that year. Since 2002, about 10 to 30 new studies started per year, with a subtle downward trend towards 2022. Because the first data was downloaded at the end of 2022, it is obvious that there are a smaller number of studies for 2023 and 2024.

**Fig. 6:**
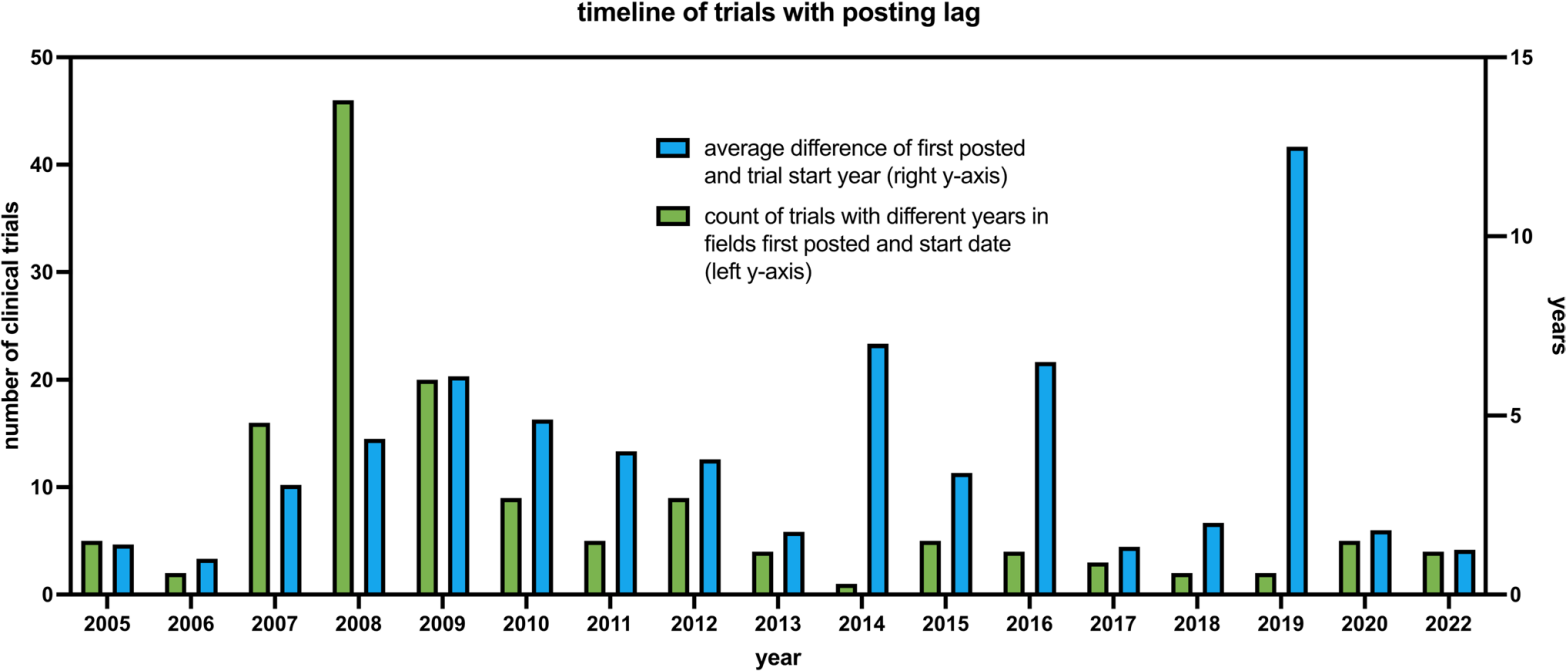
This chart shows the absolute number of clinical trials on the left *y*-axis, which had different years for the study start date and the date the study was first posted on clinicaltrials.gov. The years on the *x*-axis are based on the first posted year. On the right *y*-axis, the average difference in years between the two dates for a given year on the *x*-axis is depicted

Comparing the start dates with the dates the study was first posted on clinicaltrials.gov, we found that there were significant differences illustrated in Fig. 6. Plotting the first posted date in a similar manner to Fig. 5 (refer to Supplement Fig. 1), we found a peak in 2008. Upon closer investigation, the number of studies posted on clinical trials with different years for the date of first post and study start date peaked in 2008. Of course, sometimes these differences are negligible, for example, if study posting and start are around the end or start of the year but having a look at the average number of years between those two dates, one can clearly see a trend here as well from 2006 to 2013. The difference in years peaks in 2009 at 6.1 years.

Literature research revealed the 7th revision of the Declaration of Helsinki and the Food and Drug Administration Amendments Act (FDAAA) of 2007 as possible factors for this phenomenon (https://classic.clinicaltrials.gov/ct2/about-site/history, last accessed July 11, 2023).

The Declaration of Helsinki was first devised in 1964 to establish ethical guidelines and principles for medical research involving humans. In 2008, the 7th revision added §19 “Every clinical trial must be registered in a publicly accessible database before recruitment of the first subject,” and thus potentially promoting trials (post)registration in conjunction with stricter publication guidelines regarding trial registration by other parties (Krleza-Jerić and Lemmens 2009).

Earlier, in 2007 US Congress passed the Food and Drug Administration Amendments Act of 2007 with Sect. 801 which required stricter and more extensive trial registration in many cases and also included penalties for noncompliance (Food and Drug Administration Amendments Act of 2007 2007; https://classic.clinicaltrials.gov/ct2/about-site/history, last accessed July 11, 2023). It is plausible that trials were post-registered after the trial start date to comply with these new rules and legislations.

**Fig. 7:**
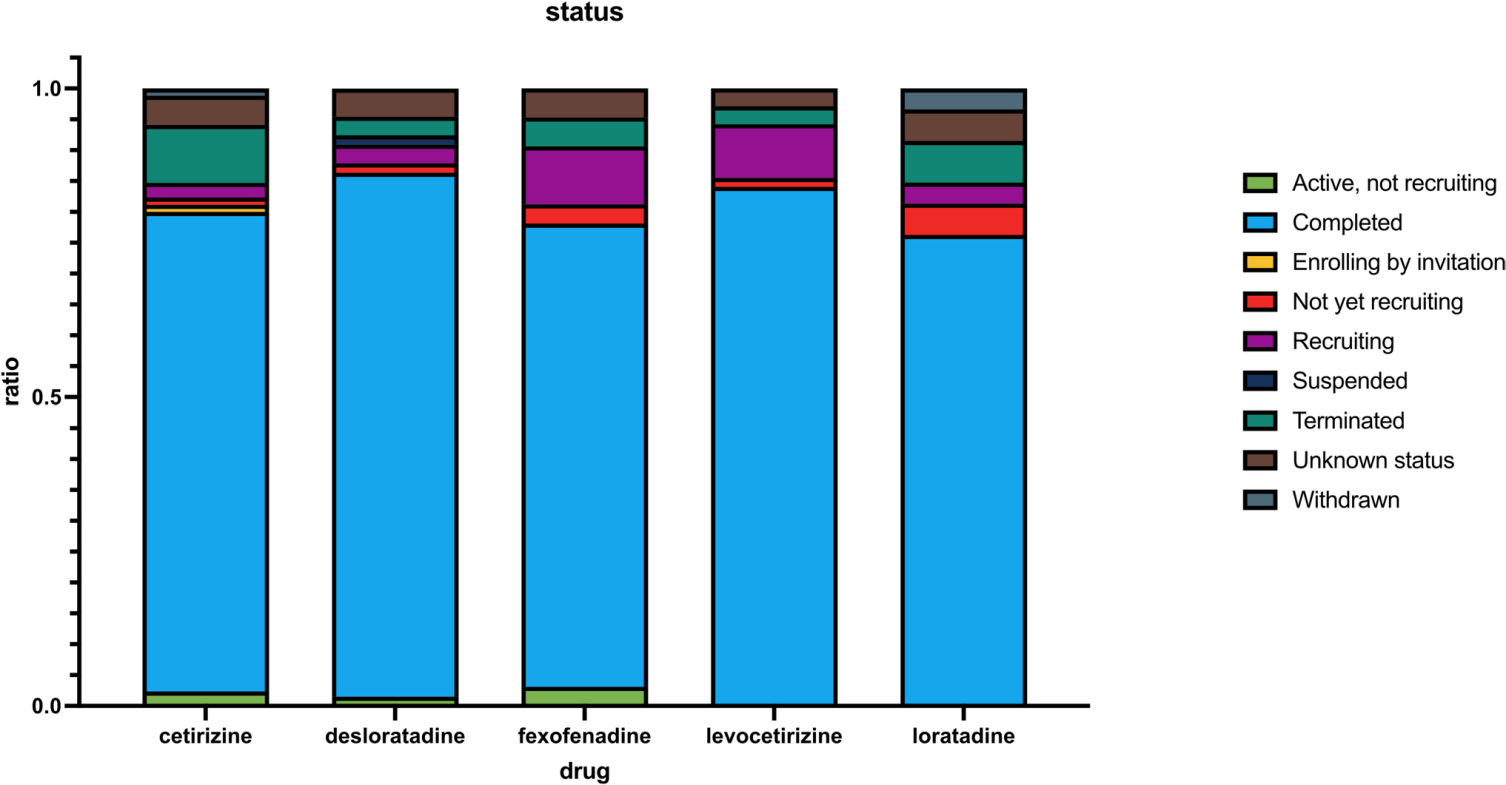
This chart shows the distribution of study status grouped by the drug name. For each drug, the distribution of categories was calculated individually as a ratio between 0 and 1

Next, we visualized the study status, which is assigned by the study authors in Fig. 7. It is evident that the majority of studies are “completed” (represented in blue); however, as we see later, this is not necessarily the case for the studies which are relevant for the topic of repurposing. Apart from active studies with various statuses (active, not recruiting, enrolling by invitation, not yet recruiting, recruiting), there are also quite a few studies that have been terminated (before completion, represented in dark green) or have a status of “unknown” (represented in brown). This means that the study has “passed its completion date and the status has not been last verified within the past 2 years.” (https://clinicaltrials.gov/study-basics/glossary, last accessed July 9, 2023).

**Fig. 8:**
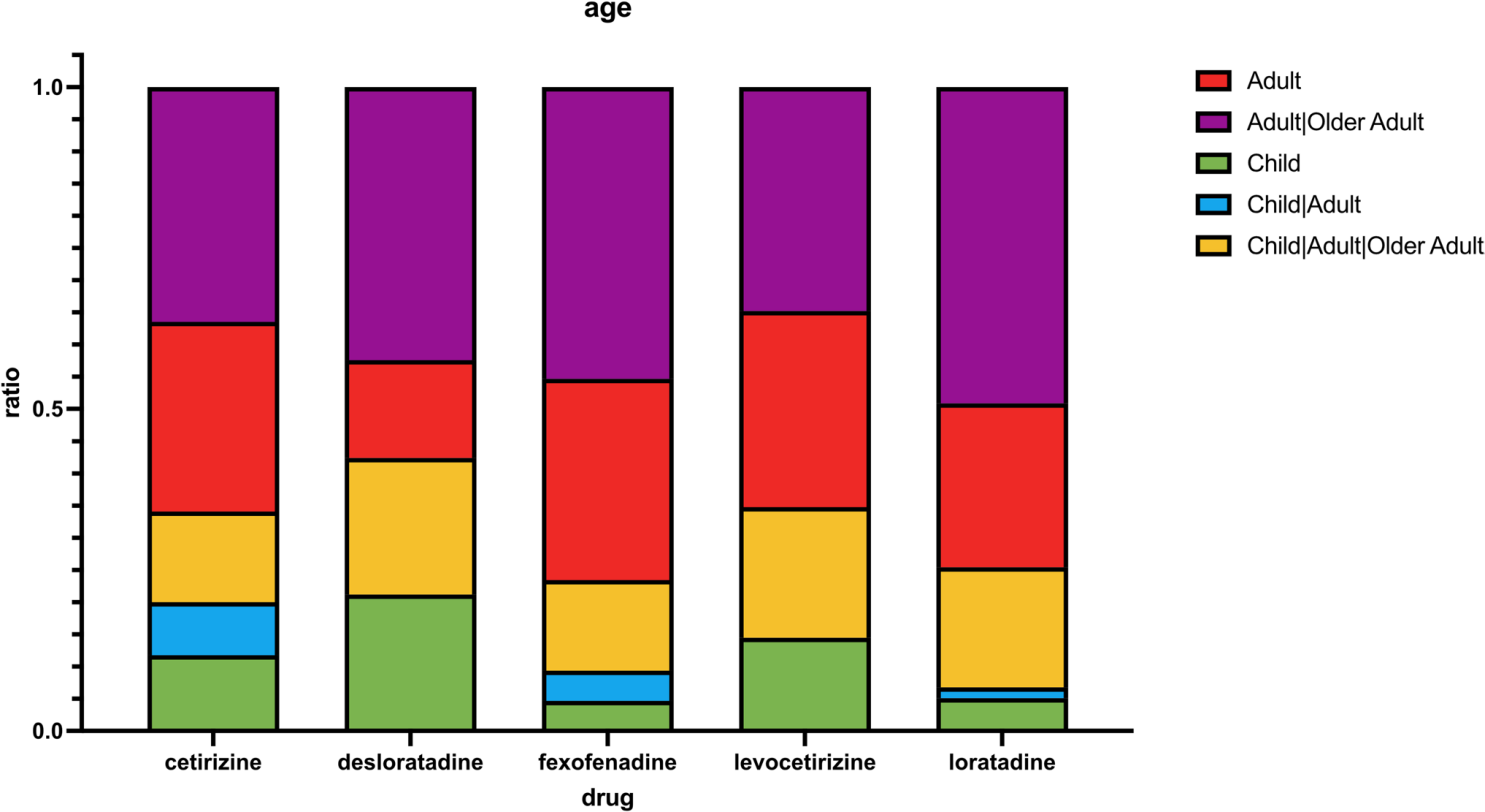
This chart shows the distribution of age groups eligible for participation in a study grouped by the drug name. For each drug, the distribution was calculated individually as a ratio between 0 and 1. Groups: child (0–17), adult (18–64), older adult (65 +)

Looking at the age groups in Fig. 8, we find that over 50% of studies are adult-only studies (red and purple), whereas only about 5 to 20% of studies are limited to children only (green). Mostly, these studies include safety evaluations or evaluations of taste, form of delivery, or preference between products. As children, clinicaltrials.gov defines ages 0–17 and 18–64 are classified as adults and everyone 65 + counts as an older adult (https://clinicaltrials.gov/study-basics/glossary, last accessed July 9, 2023).

Furthermore, analysis and visualization (refer to Supplement Fig. 2) of the sex eligible to participate in a study revealed that male- or female-only studies are far less common than studies for all. In 90–95% of studies, both female and male candidates are eligible (of course, other eligibility criteria are neglected). Female-only studies were the least common, between 0 and 2%.

Moreover, we attempted to visualize the assigned use cases for the H_1_R antagonists in conjunction with the information on whether these studies were interesting for further investigation regarding repurposing.

**Fig. 9:**
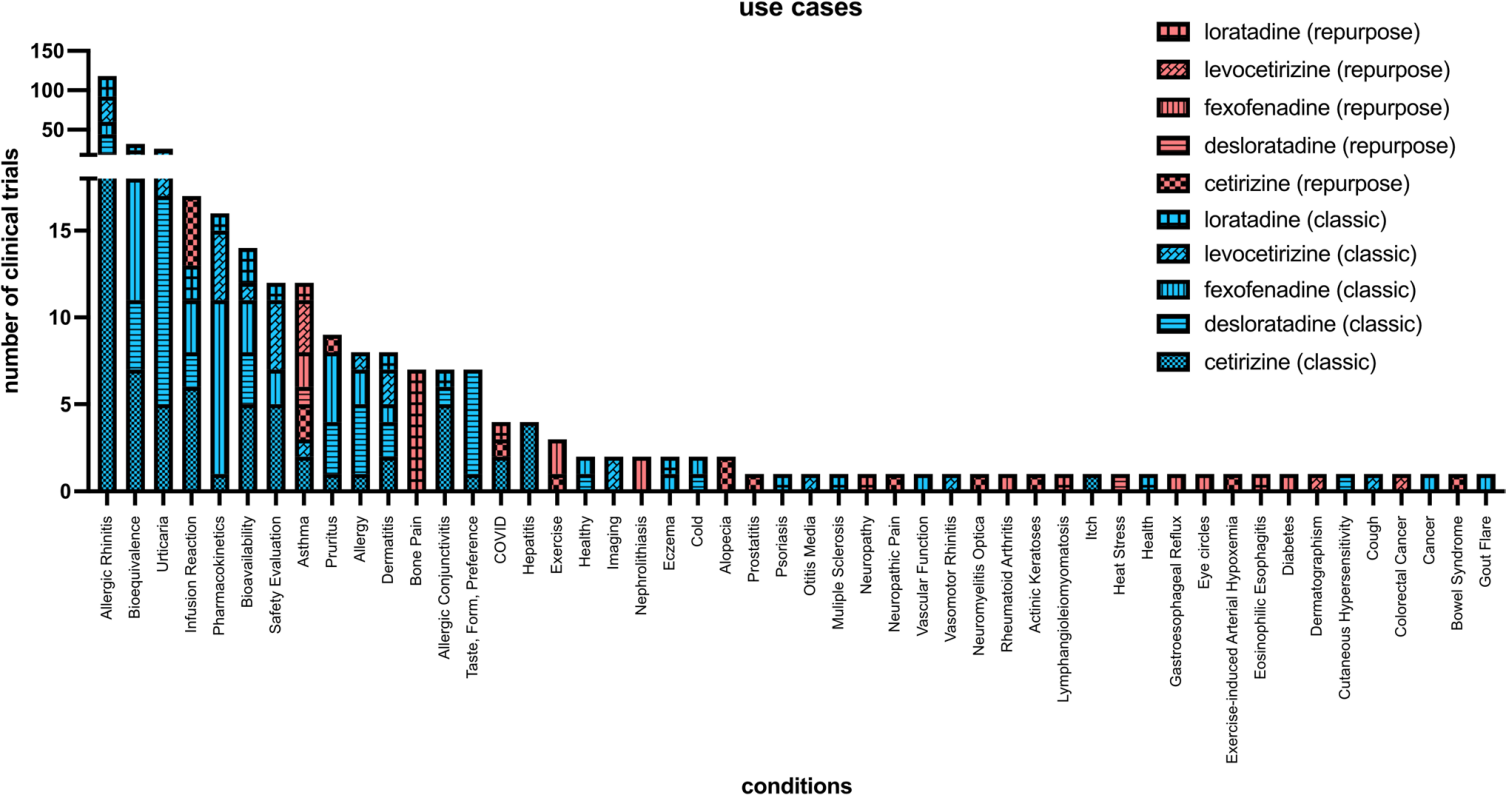
This chart visualizes the absolute number of studies for the use cases of H_1_R antagonists we manually assigned, color- and pattern-coded by drug name, and repurposing/classic use. Studies that may be interesting for repurposing are color-coded in red. Because there were many studies for the first use cases, the *y*-axis is discontinuous with different scaling for each part

As depicted in Fig. 9, there were a lot of studies for the conditions of allergic rhinitis, urticaria, and many studies about bioequivalence. “Repurposing studies” only start to appear in the topics with fewer numbers of trials and are most prevalent with the topics that are only represented by one study.

**Fig. 10:**
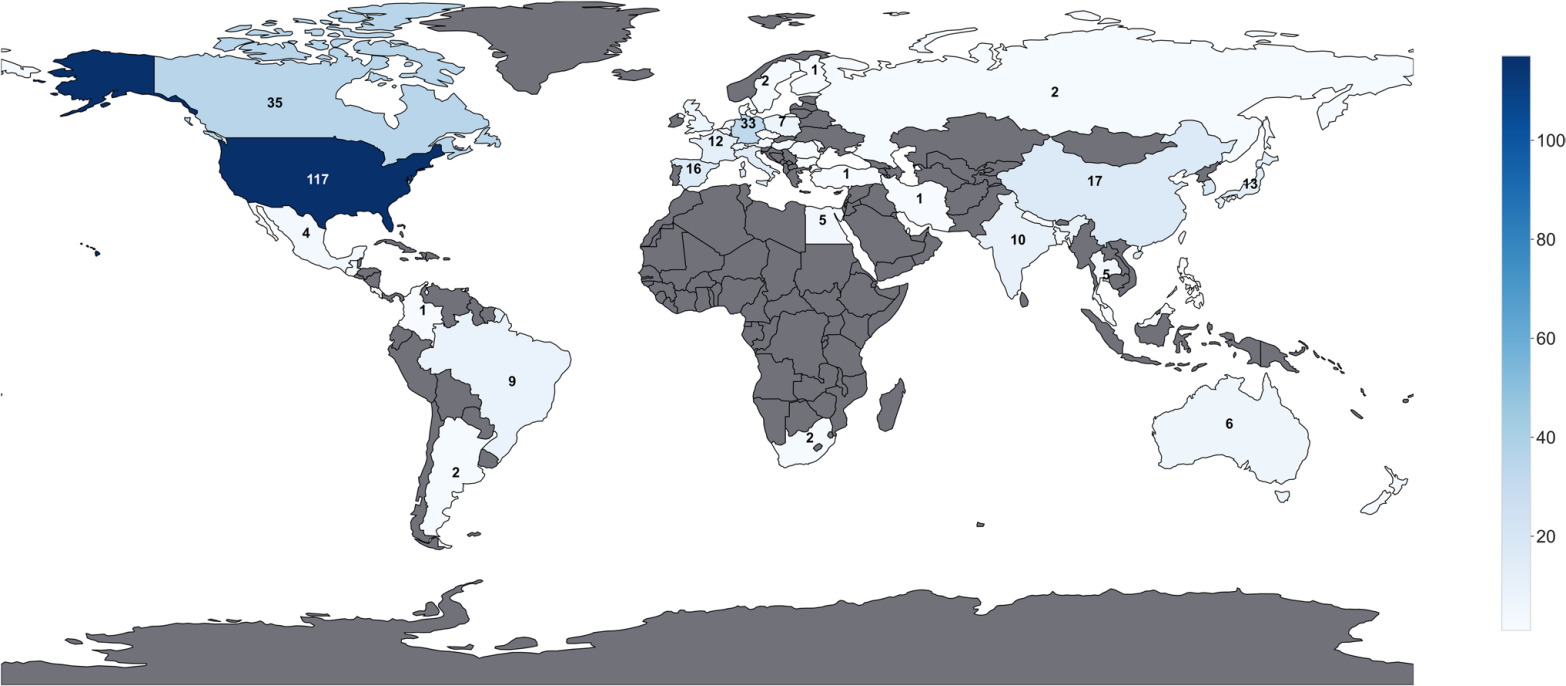
This chart shows the absolute number of studies that have at least one location in the given country. Countries that do not have any study locations in our dataset are depicted in grey. For improved legibility, text labels are only displayed for countries bigger than a certain threshold area

Lastly, the locations of studies were plotted on a map using Matplotlib and GeoPandas. Here, we tried to recreate the map view found on clinicaltrials.gov (the old website version) with the downloaded data. To achieve a similar result, the number of studies for a country was only increased by one for each study, even if this study had multiple locations in the given country. The result can be seen in Fig. 10. The USA has the most studies with location in the country (117), followed by Canada (35) and Germany (33).

Coming back to the way of study aggregation for a country described above, the individual count of study *locations* would have accumulated to about 700 for the USA, as an example for illustration.

### Publication und bias

In Fig. 9, we illustrated the number of repurposing studies in red. However, not all of those studies were completed and had results available.

**Fig. 11:**
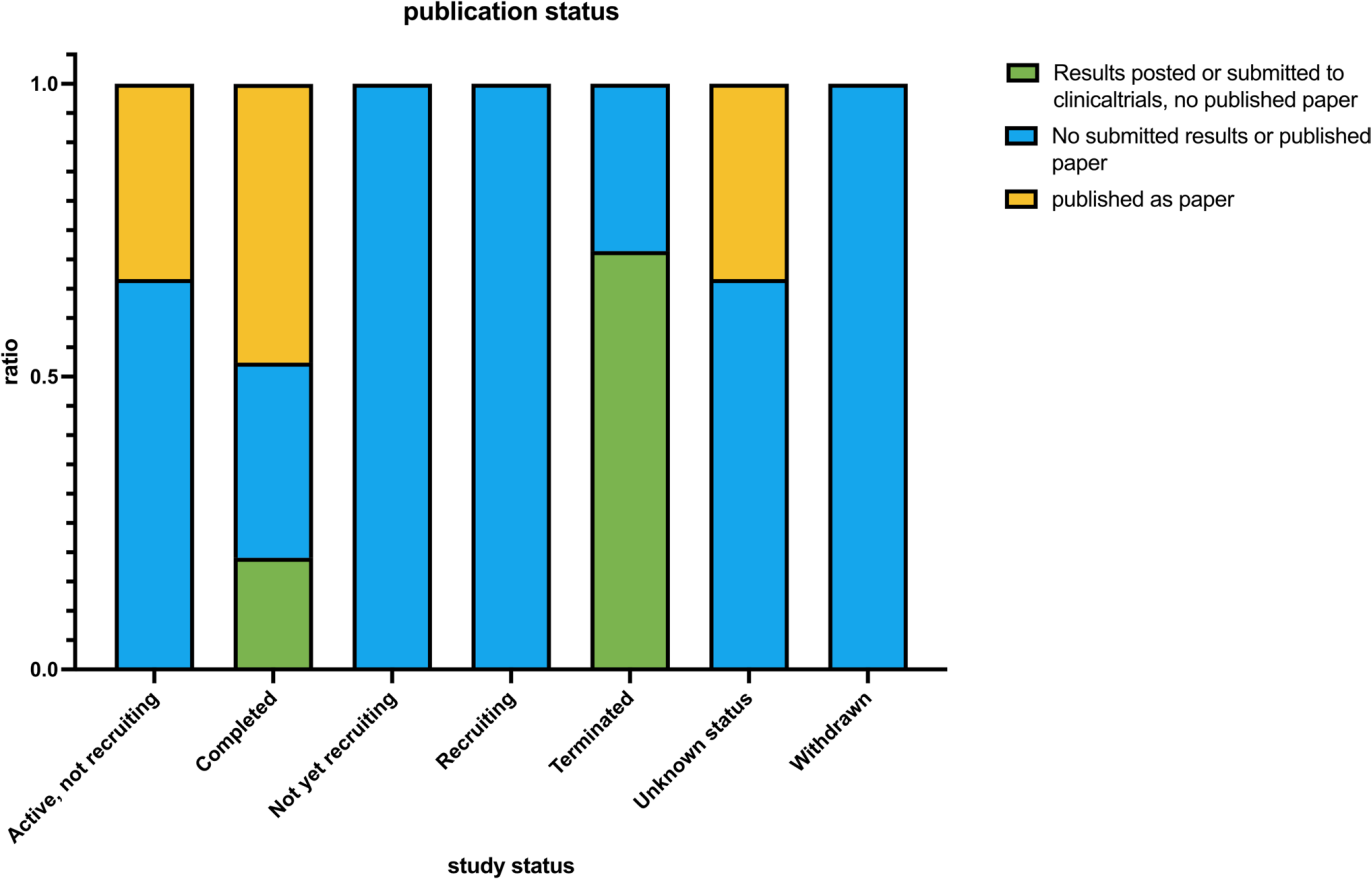
This chart depicts the distribution of publications as a paper, submission of study results on clinicaltrials.gov, or no publication of results grouped by study status. Only the studies that were marked as repurposing were included here

In fact, Fig. 11 shows that only 10 out of 22 studies with a status of “completed” were published as a paper (about 45.5%, represented in yellow). Additionally, we found two other studies that were published with a status of “active, not recruiting” and “unknown.” Since the data download, the “active, not recruiting” study changed its status to “completed” and was subsequently published. For about 58% of these studies, there were “positive” results.

It is also noteworthy that a majority of terminated studies had results submitted to clinicaltrials.gov without publication of a paper.

### Repurposing studies: an overview

Given the challenges described in the previous section regarding the low number of publications of studies with repurposing characteristics, we initially had very little literature to work with.

**Fig. 12:**
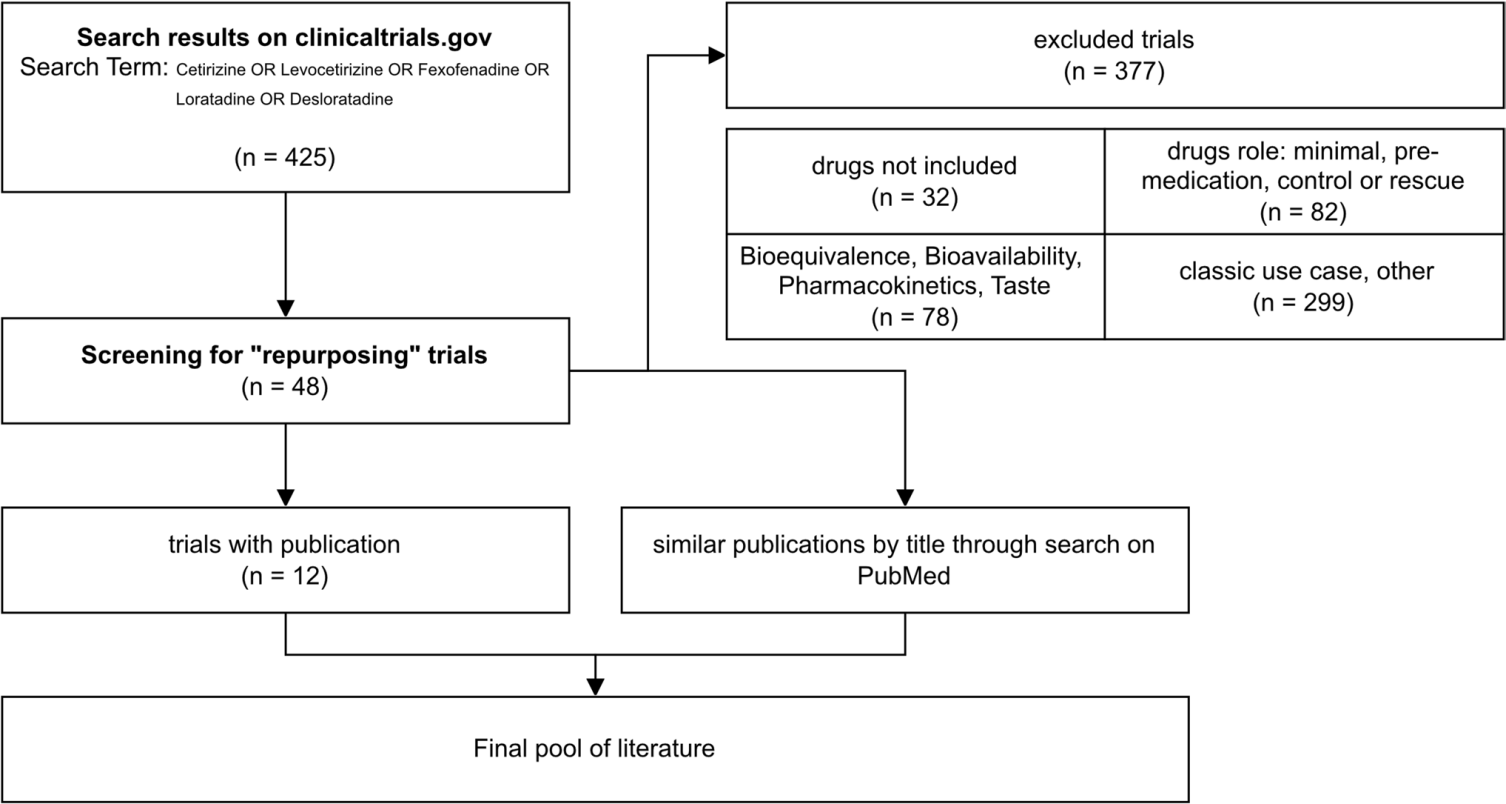
This flowchart shows the absolute number of clinical trials for selected categories during our process of filtering for relevant studies with regard to repurposing. Excluded trials include all trials not marked as repurposing. “classic use case, other” includes all excluded trials that were assigned use case other than bioequivalence, bioavailability, pharmacokinetics or taste [form and preference]

However, the systematic analysis of the trials still revealed potential areas for repurposing, even though results were not or not yet published. This allowed us to identify publications with similar or sometimes even identical topics to the use case studied in the trials to gain an overview of potential repurposing. Figure 12 illustrates the process as a flow chart: Out of 425 studies that matched our search term, roughly 50 were interesting for the topic of repurposing, and for only 12 we were able to find published results (refer to Table 2 for an overview of these 12 papers) but through augmentation with similar topic publications on PubMed, a final pool of literature was procured.

**Table 2:** Overview of published studies we considered interesting in the context of repurposing

#	Publication	H_1_R antagonists	Indication	Study design	Result	Additional remarks
1	Pardo G, Boutwell C, Conner J, et al. (2010) Effect of oral antihistamine on local injection site reactions with self-administered glatiramer acetate. J Neurosci Nurs 42:40–46. https://doi.org/10.1097/jnn.0b013e3181c71ab7	Cetirizine (10 mg)	Local injection site reaction	Randomized, double-blind, placebo-controlled with parallel arms	“Oral antihistamine treatment prior to self-injection of glatiramer acetate did not result in a statistically significant reduction in the number of LISRs at 5 min after injection”	“it is possible that an effect might have been seen if cetirizine had been taken several hours prior to the glatiramer acetate injection instead of 30 min”
2	Davis BE, Illamperuma C, Gauvreau GM, et al. (2009) Single-dose desloratadine and montelukast and allergen-induced late airway responses. Eur Respir J 33:1302–1308. https://doi.org/10.1183/09031936.00169008	Desloratadine (5 mg)	Asthmatic response after allergen challenge	Randomized, double-blind, placebo-controlled with crossover arms	“Desloratadine reduced the response by 43%, montelukast reduced it by 71% and the combination completely blocked the response”	Small sample size This might not be the best example of a study for repurposing, but it is one of the few studies regarding asthma and potential repurposing published. Here, the limitation of “subjective” selection of repurposing studies can be seen as in some cases, studies are just on the threshold of repurposing
3	Moukharskaya J, Abrams DM, Ashikaga T, et al. (2016) Randomized phase II study of loratadine for the prevention of bone pain caused by pegfilgrastim. Support Care Cancer 24:3085–3093. https://doi.org/10.1007/s00520-016-3119-0	Desloratadine (10 mg)	Prevention of bone pain caused by pegfilgrastim	Randomized, quadruple-blind, placebo-controlled with parallel arms	“This study demonstrated that prophylactic administration of the antihistamine loratadine at standard dose does not decrease the incidence of significant pegfilgrastim-induced bone pain”	Limitation (as mentioned by the authors): Use of analgetics was permitted, reliance on a questionnaire which is also based on the patient’s recall, small sample size, bone pain may improve with chemotherapy cycles (here, observation with loratadine started only after bone pain was observed)
4	Kirshner JJ, McDonald MC, Kruter F, et al. (2018) NOLAN: a randomized, phase 2 study to estimate the effect of prophylactic naproxen or loratadine vs no prophylactic treatment on bone pain in patients with early-stage breast cancer receiving chemotherapy and pegfilgrastim. Support Care Cancer 26:1323–1334. https://doi.org/10.1007/s00520-017-3959-2	Desloratadine (10 mg)	Prevention of bone pain caused by pegfilgrastim	Randomized, open-label with parallel arms (women only)	“Patient-reported bone pain [was] consistently lower in the naproxen and loratadine groups than in the no prophylaxis group”, however mostly not statistically significant “Given its tolerability, its ease of administration, and the consistent reductions in patient-reported bone pain […] [loratadine] should be considered for patients receiving chemotherapy and pegfilgrastim”	Open-label trial with no placebo control; “data on bone pain were captured by asking patients about any AEs they had experienced since their last clinic visit,” other medications for pain were allowed but documented
5	Amin M, Desai M, Trinkaus K, et al. (2019) Phase II trial of levocetirizine with capecitabine and bevacizumab to overcome the resistance of antiangiogenic therapies in refractory metastatic colorectal cancer. J Gastrointest Oncol 10:412–420. https://doi.org/10.21037/jgo.2019.02.01	Levocetirizine (5 mg)	Overcoming anti-angiogenic therapy resistance in patients with refractory metastatic colorectal cancers	Randomized, open-label with parallel arms	The addition of levocetirizine was well tolerated but an ability to overcome resistance to anti-angiogenic therapy could not be demonstrated	Open-label trial with no placebo control, both arms included levocetirizine but at different time points in treatment
6	Vanaman Wilson MJ, Jones IT, Wu DC, Goldman MP (2017) A randomized, double-blind, placebo-controlled clinical trial evaluating the role of systemic antihistamine therapy for the reduction of adverse effects associated with topical 5-aminolevulinic acid photodynamic therapy. Lasers Surg Med 49:738–742. https://doi.org/10.1002/lsm.22682	Cetirizine (10 mg)	Reduction of adverse effects associated with photodynamic therapy for actinic keratosis	Randomized, double-blind, placebo-controlled with parallel arms	“Oral H1 antihistamines did not mitigate the inflammatory adverse effects patients experience after ALA-PDT, nor did they impair the efficacy of ALA-PDT in reducing actinic keratoses. Healing, cosmetic improvement, and subject satisfaction were similarly not affected by the use of antihistamines”	Small sample size
7	Katz Sand I, Fabian MT, Telford R, et al. (2018) Open-label, add-on trial of cetirizine for neuromyelitis optica. Neurol Neuroimmunol Neuroinflamm 5:e441. https://doi.org/10.1212/NXI.0000000000000441	Cetirizine (10 mg)	Relapse prevention of neuromyelitis optica with cetirizine as add-on therapy	Open-label, single arm	It could be seen that the annualized relapse rate dropped significantly compared to a pre-study without cetirizine	Being a pilot study, there are many limitations to consider: open label, lack of control group, small sample size
8	Costa ACC, Yamamoto PA, Lauretti GR, et al. (2020) Cetirizine Reduces Gabapentin Plasma Concentrations and Effect: Role of Renal Drug Transporters for Organic Cations. J Clin Pharmacol 60:1076–1086. https://doi.org/10.1002/jcph.1603	Cetirizine (20 mg)	Exploration of the interaction between cetirizine and gabapentin for treatment of neuropathic pain	Non-randomized, open-label with crossover arms	“Coadministration of CTZ led to a reduction in plasma GBP concentration and in pain attenuation. These alterations are in the opposite direction from that expected for the inhibition of GBP renal active transport by CTZ”	Also heavy focus on in vitro experiments in addition to the clinical trial itself
9	Holmes JP, Peguero JA, Garland RC, et al. (2021) Intravenous Cetirizine vs Intravenous Diphenhydramine for the Prevention of Hypersensitivity Infusion Reactions: Results of an Exploratory Phase 2 Study. J Infus Nurs 44:315–322. https://doi.org/10.1097/NAN.0000000000000444	Cetirizine (10 mg IV)	Prevention of Infusion associated reactions	Randomized, double-blind with parallel arms	“IV cetirizine (10 mg) is an effective and safe alternative to IV diphenhydramine (50 mg) in the prevention of hypersensitivity infusion reactions”	Small sample size
10	Beaucage-Charron J, Gaudet L, Lamothe S, et al. (2022) A randomized double-blind feasibility study comparing cetirizine and diphenhydramine in the prevention of paclitaxel-associated infusion-related reactions: the PREMED-F1 study. Support Care Cancer 30:3389–3399. https://doi.org/10.1007/s00520-021-06734-4	Cetirizine (10 mg oral)	Prevention of infusion-associated reactions/feasibility study	Randomized, double-blind, placebo-controlled with parallel arms	“Cetirizine produced less drowsiness when used as premedication than diphenhydramine. Given the infrequency of paclitaxel-related IRR found in our setting, especially severe events requiring medical intervention, consideration for a large multi-center non-inferiority trial using a predetermined non-inferiority margin is warranted.”	This is a feasibility study to see if a larger trial is warranted and possible
11	Van der Stede T, Blancquaert L, Stassen F, et al. (2021) Histamine H1 and H2 receptors are essential transducers of the integrative exercise training response in humans. Sci Adv 7:eabf2856. https://doi.org/10.1126/sciadv.abf2856	Fexofenadine (540 mg)	The role of histamine H_1_ and H_2_ receptors in training adaption	Randomized, double-blind, placebo-controlled with parallel arms	“The sustained elevation of muscle perfusion after acute interval exercise was severely reduced when H1/H2 receptors were pharmaceutically blocked. Our work suggests that histamine H1/H2 receptors are important transducers of the integrative exercise training response in humans, potentially related to regulation of optimal post-exercise muscle perfusion.”	This study is not necessarily a case of repurposing of H_1_R antagonists for new clinical indications but rather as a tool to understand pathophysiology
12	Bassiouny EA, El-Samanoudy SI, Abbassi MM, et al. (2022) Comparison between topical cetirizine with minoxidil versus topical placebo with minoxidil in female androgenetic alopecia: a randomized, double-blind, placebo-controlled study. Arch Dermatol Res. https://doi.org/10.1007/s00403-022-02512-2	Cetirizine (topical 1%, 1 mL) with minoxidil (5%, 1 mL)	Androgenetic alopecia in females	Randomized, double-blind, placebo-controlled with parallel arms (women only)	The addition of cetirizine had some statistically significant improvements (especially in patients’ self-assessment) while displaying no safety concerns	“Limitations of this study included the absence of a cetirizine arm because by the time the study started there was only one paper about the efficacy of topical cetirizine in AGA”

Repurposing use cases that presented themselves more than once in our dataset and had published results of some form were asthma, infusion-associated reactions (IARs), G-CSF-associated bone pain, alopecia, and COVID-19.

There were other topics that warrant a closer look as well. They, however, were not as prominent as those mentioned above.

### Asthma

Asthma and H_1_R antagonists are an old topic. Recent reviews mention that H_1_R antagonists were first used in the treatment of asthma in the 1940s, but nowadays, they are only believed to be effective in subtypes of asthmatics with allergic characteristics (Yamauchi and Ogasawara 2019). Historically, the use of earlier-generation H_1_R antagonists resulted in airway dilation, and therefore, the H_1_ receptor was believed to be clinically useful in asthma treatment. However, the newer and more selective generation H_1_R antagonists revealed that these earlier dilatory effects were most likely caused by M_x_R antagonism of first-generation antagonists (Yamauchi and Ogasawara 2019). Additionally, mast cells were believed to be the primary mediator, while nowadays, asthma is “viewed as a multifactorial chronic inflammatory condition” with many contributing factors (Church 2017).

Trials in the clinicaltrials.gov database oftentimes dated back 10 or even 20 years but very rarely could we match published results to these studies. When we did, results were mostly positive using a combination of both the cysteinyl leukotriene receptor antagonist montelukast and H_1_R antagonists. For example, “Montelukast was non-significantly better than desloratadine but not as effective as the combination. There was a trend towards a decrease in airway responsiveness following montelukast and combination.” (reduction in airways response for desloratadine 43%, montelukast 71%, combination blocked the response completely, *n* = 10) (Davis et al. 2009). Another study found that the combination of montelukast with loratadine was comparable to the combination of montelukast and the glucocorticoid budesonide (Wei et al. 2019), while yet another study found that the combination of levocetirizine and montelukast had a positive impact on patients with asthma *and* allergic rhinitis (reduction in mean daytime nasal symptom score − 0.98 vs. − 0.81 for montelukast alone; *p* = 0.045) (Kim et al. 2018). It is not surprising that a positive impact could be seen as allergic rhinitis was involved. The effect of the H_1_R antagonists itself on asthma remains questionable.

“The nose is the guardian of the lung,” and therefore, treatment of allergic rhinitis with H_1_R antagonists might be beneficial for asthma and its development (Church 2017); however, in terms of repurposing of H_1_R antagonists against asthma itself, there is little evidence nowadays.

### Infusion-associated reactions

Infusion-associated reactions (IARs) are common but also unpredictable for patients receiving chemotherapy. They need to be minimized for the patient to receive the best treatment possible. Potential improvement in preventing IARs could be newer generations of H_1_R antagonists. H_1_ receptor antagonists are considered because of the involvement of the H_1_ receptor in urticaria, smooth muscle contraction, and vasoconstriction. (ALMuhizi F et al. 2022). Currently, diphenhydramine intravenous injection (a first-generation H_1_R antagonist) seems to be the standard as a premedication for IARs and the replacement of diphenhydramine with cetirizine was a fairly popular research topic on clinicaltrials.gov. While an earlier study (NCT00240032, completed in 2006) looked at the effect of oral cetirizine on the injection site of chemotherapy with no statistically significant difference between placebo and cetirizine (Pardo et al. 2010), more recent trials are focused on the replacement of diphenhydramine.

In NCT04237090, the feasibility of a clinical trial was tested and deemed acceptable. Here, cetirizine produced less drowsiness and seemed to be a replacement worth looking into further (Beaucage-Charron et al. 2022).

An earlier study already believed cetirizine to be a “viable substitute” but saw the need for further explorations due to limitations of the study, like a small sample size, a short duration, or differences in infusion frequency. Reports of adverse effects regarding the antihistamines were given subjectively by the nursing staff; however, they did not report any adverse effects while using cetirizine (Durham et al. 2019).

Holmes et al. (2021) came to a similar conclusion: Although they also had a small sample size, intravenous cetirizine was shown to be as effective as diphenhydramine while being associated with less adverse reaction and drowsiness. It may also be even more beneficial for older patients whose clearance of diphenhydramine may be inadequate.

### G-CSF-related bone pain

G-CSFs (granulocyte-colony stimulating factors), such as filgrastim and pegfilgrastim, are used in chemotherapy to treat secondary (adverse) effects, namely neutropenia, by causing proliferation of myeloid cells in the bone marrow and intervening in the bone metabolism. This, however, can cause severe pain and is believed to be associated with histamine release as one of the factors involved, although the exact mechanism is still unclear (Moore and Pellegrino 2017). Higher histamine levels were associated with inflammatory reactions due to G-CSF, which may lead to neuropathic pain or cause edema formation within the bone (Lambertini et al. 2014).

Because of the connection to histamine, H_1_R antagonists are being tested for treatment.

In a first case report from 2015 (Romeo et al. 2015), the use of loratadine completely alleviated the bone pain that was induced through pegfilgrastim in a 67-year-old female patient with ovarian cancer. Most notably, the pain was described as 10/10 on the Likert pain scale and was gone completely after premedication with loratadine before pegfilgrastim treatment.

Larger studies showed mixed results, oftentimes with no statistical advantage of loratadine: Moukharskaya et al. (2016) studied 213 patients and assessed the incidence of bone pain with loratadine compared to placebo and found no significant difference. Patients were permitted to use non-steroidal anti-inflammatory drugs, however. The authors discuss that patients with lower pain thresholds were also included in the study.

Gavioli and Abrams (2017) used a combination of loratadine and famotidine as a double histamine blockade and reported positive results. Here, sample sizes also were small (*n* = 17), and the authors say that “determining the clinical significance of [double histamine blockade] is challenging based on the retrospective nature of the study,” but the bone pain seemed to be alleviated (average difference of 1.21 in pain score; *p* = 0.019).

In a larger study on 600 patients (Kirshner et al. 2018), the occurrence of bone pain in women with breast cancer was investigated. In this open-label trial, patients received either no treatment, naproxen, or loratadine to prevent bone pain. While differences between treatment arms were mostly not statistically significant at the 5% level, “patient reported bone pain was consistently lower in the naproxen and loratadine groups than in the no prophylaxis group by every measure” (for example, mean patient-reported bone pain on a scale of 1–10 in cycle 1 with no prophylaxis 3.9, with naproxen 3.3, and with loratadine 3.0). Patients were allowed to take additional medication, which was more often the case in the no prophylaxis group. Kirshner et al. conclude that “given its tolerability, its ease of administration, and the consistent reductions in patient-reported bone pain observed in this study, treatment with five days of once daily loratadine in each chemotherapy cycle should be considered for patients receiving chemotherapy and pegfilgrastim.”

### Alopecia

Androgenetic alopecia (AGA) is a common chronic disorder in men and women characterized by progressive hair loss. The only FDA-approved treatment options are topical minoxidil and oral finasteride or hair transplants (Chen et al. 2022). Additionally, the *off-label* use of dutasteride which, similarly to finasteride, inhibits the 5-alpha-reductase that converts testosterone to dihydrotestosterone (DHT) is a possible treatment option. Compared to finasteride, it seems to display a higher efficacy (Bajoria et al. 2023).

The exact pathogenesis of AGA is unknown but high DHT levels, increased androgen receptor expression, and prostaglandins seem to play a role (prostaglandin D2 (PDG2) inhibits growth, prostaglandin E2 (PGE2) does the opposite). Cetirizine has been shown to inhibit PGD2 and increase PGE2 (Chen et al. 2022). Therefore, cetirizine may be an effective treatment option or alternative. In all studies and trials, cetirizine was applied topically.

A study comparing cetirizine to minoxidil found that cetirizine was a viable medication to treat AGA. However, minoxidil showed bigger effects (physician assessment of hair density after 16 weeks cetirizine vs. minoxidil: slight increase 33% vs. 25%, no difference from baseline 50% vs. 75%, slight decrease 17% vs. 0%; *n* = 30). Nonetheless, the use of cetirizine showed potential, and no adverse reactions were reported. The authors came to the conclusion that considering cetirizine for AGA treatment seems like a “useful idea” (Hossein Mostafa et al. 2021). The study was limited to male participants and ages between 18 and 49 were included.

Another more recent placebo-controlled study showed similar results with male participants in a similar age group (22 to 55 years): The difference in improvement was significant; however, photographic casement yielded mixed results. Second to no improvement (*n* = 17), there was mild improvement in *n* = 10 out of 30 cases. Placebo groups did not show any improvement, though (Zaky et al. 2021).

In the published results of trial NCT04481412, female patients aged 20 to 50 with AGA were investigated. Here, however, cetirizine was used as an addition in treatment in conjunction with minoxidil. The authors explain that “Limitations of this study included the absence of a cetirizine arm because by the time the study started, there was only one paper about the efficacy of topical cetirizine in AGA,” and they therefore did not want to include a cetirizine-only arm. The addition of cetirizine had some statistically significant improvements (especially in patients’ self-assessment) while displaying no safety concerns (Bassiouny et al. 2022).

A recent systematic review on the use of topical cetirizine for AGA from 2022 came to the following conclusion: “In comparison with topical minoxidil, topical cetirizine appears to be less effective for improving total and vellus hair density, but it might have a longer-lasting effect. Furthermore, cetirizine might be as effective as minoxidil in improving hair diameter.” The authors also pointed out that cetirizine, while maybe not as effective in some areas, may be a good choice for patients who display negative responses to minoxidil because of its low cost and its safety profile (Chen et al. 2022).

### COVID-19

Whereas previous use cases seemed to be centered around one of the investigated H_1_R antagonists, studies and trials for coronavirus disease 2019 (COVID-19) included multiple. It is possible that H1 receptor antagonists may be beneficial in mitigating lung inflammation by mast cell stabilization and cytokine inhibition through H_1_R (Qu et al. 2021).

NCT04836806 tried to study the effect of cetirizine in combination with famotidine, but the study was withdrawn due to a lack of enrollment and funding. Previously, there had been a paper regarding this topic presenting a physician-sponsored study. It was open-label and there was no placebo control. Comparisons to cohorts from other regions and preliminary results from the same medical center seemed to indicate that dual-histamine receptor blockade might reduce severity (e.g., 16.4% vs. 41.7% intubations, 11 vs. 18 days of hospitalization; however “not deemed sufficient for comparative statistical analysis”). Even though the study is limited, the authors explain that due to the severity of the COVID-19 pandemic, this attempt for a “rational repurposing” has been made to provide a proof-of-concept off-label treatment which resulted in the implementation of dual histamine blockade in the vast majority of severe COVID cases in this study’s medical center (Hogan Ii et al. 2020).

A 2021 review asking whether the use of antihistamines in the COVID-19 treatment paradigm is a “hype or [a] hope” came to the conclusion that H_1_ and H_2_ blockade or even better the combination of both may be beneficial for the management of COVID-19. Cetirizine may even be effective in suppressing virus replication (Al-Kuraishy et al. 2021).

Another treatment approach was the combination of levocetirizine and montelukast. Here, again, there was no placebo arm, and at first, cetirizine and levocetirizine were “interchangeably” but the FDA accepted the data as a proof of concept to justify further research using the combination (e.g., symptom resolution within 7 days with levocetirizine and montelukast vs. 10–14 days without) (May and Gallivan 2022).

In a more theoretical in vitro approach, the effect of desloratadine and loratadine on the integral membrane protein angiotensin-converting enzyme-2 (ACE-2) was studied. ACE-2 seems to be important for the virus to enter the cell. By binding with ACE2, most notably desloratadine (stronger bond than loratadine) may be able to prevent the virus from entering the cells in the first place (viral entry ratio was reduced from 1 to 0.68 ± 0.07 for loratadine and 0.23 ± 0.10 for desloratadine) (Hou et al. 2021).

While there were proof-of-concept studies to use H_1_R antagonists to improve COVID-19 treatment paradigms, all these studies were quite limited and lacked placebo control. The addition of antihistamines seems reasonable and may even have other beneficial effects like the ACE2 interaction of desloratadine. However, there seems to be a lack of placebo-controlled studies, and current research seems to be the result of small tests conducted during the pandemic.

### Other indications

As mentioned above, there were other topics and use cases that were published but only appeared once in our clinical trials dataset.

One published trial (NCT01722162) looked at levocetirizine to enhance chemotherapy in patients with refractory colorectal cancer. The idea was that levocetirizine had been shown to inhibit IL-8, which had been associated with angiogenesis and resistance to antiangiogenic therapies.

Combined with capecitabine and bevacizumab, levocetirizine was administered 7 days prior or 7 days after chemotherapy to have two different arms.

Results did show similar progression-free survival rates than other treatment plans and IL-8 levels were lower in patients with stable disease.

The addition of levocetirizine was well tolerated, but an ability to overcome resistance to anti-angiogenic therapy could not be demonstrated (Amin et al. 2019).

Neuromyelitis Optica (NMO) is an inflammatory disease of the CNS. A pilot study (NCT02865018) incorporated daily cetirizine into the treatment regimen of patients who had previously suffered from an NMO episode. Cetirizine was selected as a potential treatment option because of its properties as an eosinophil stabilizer which, other studies had shown, seems to be of importance for local inflammatory events of NMO.

It could be seen that the annualized relapse rate dropped significantly compared to a pre-study without cetirizine (0.4 ± 0.80 before cetirizine, 0.1 ± 0.24 after cetirizine; *p* = 0.047). However, this being a pilot study, there are many limitations to consider: open label, lack of control group, and small sample size.

In general, cetirizine seems like a promising treatment option for NMO but further and more extensive trials are needed (Katz Sand et al. 2018).

A study (NCT03047278) tried to evaluate the interaction between gabapentin (GBP) and cetirizine in patients with neuropathic pain. GBP is an organic cation drug used to treat neuropathic pain and it is eliminated renally. This study tried to find a connection between the inhibition of transporters potentially responsible for GBP renal secretion by cetirizine with the hypothesis that cetirizine might decrease GBP renal secretion by OCT2 inhibition and therefore may result in higher pain attenuation. They, however, found that clinically, the coadministration of cetirizine led to a reduced plasma concertation of GBP, which is the opposite they expected (Costa et al. 2020).

There were multiple studies (NCT05095311, NCT03192488, NCT04450134, NCT05131555) that had the general topic of histamine and exercise and training performance. The one published study on the effect of histamine 1 and 2 receptors on exercise training suggests that they are essential for the adaptions associated with it. Blockage of histamine receptors reduced muscle perfusion and vascular function (Van der Stede et al. 2021). While this may not be a use case for drug repurposing in the traditional sense to provide new treatment options, it shows that the use of H_1_R antagonists can also be helpful in revealing pathophysiological insights.

In a small case report of five patients, antihistamines seemed to have an effect on irritable bowel syndrome (IBS) with diarrhea (Hassoun et al. 2019), which inspired the authors to start a larger clinical trial (NCT04612803) which yet has to yield results.

There has also been a report about the combination of cetirizine with famotidine (also mentioned in the publication of the COVID-19 treatment paradigm using these two antihistamines (Hogan Ii et al. 2020)) which came to the conclusion that the combination of H_1_R and H_2_R antagonists may be an effective treatment (Mohammadi et al. 2018 [abstract only]).

There is an ongoing study on the effects of loratadine on lymphangioleiomyomatosis (LAM), a rare disease characterized by progressive cystic lung destruction. Previous studies suggested that the combination of loratadine with SOC (rapamycin) may prove to be beneficial as LAM is associated with high levels of histamine and VEGF (Herranz et al. 2021).

### Non clinicaltrials.gov related topics

During literature research, we found publications with topics that did not come up in our clinicaltrials dataset, but which are relevant for repurposing. To give a comprehensive overview, we will present these papers briefly.

Fritz et al. found that (des)loratadine seemed to have a positive impact on cancer survival. In one study (2020b), the authors analyzed a nationwide register in Sweden regarding prescribed antihistamine use and breast cancer diagnoses (61,627 women). They found that the usage of loratadine (hazard ratio (HR) = 0.80) and desloratadine (HR = 0.67) correlated with an improved survival rate. They could not find this correlation with four other H_1_R antagonists (including cetirizine (HR = 1.07) and fexofenadine (HR = 0.89)). Ebastine showed effects as well, although less than (des)loratadine.

Similarly, they found that loratadine (HR = 0.50) and desloratadine (HR = 0.46) were associated with improved survival in comparison to non-use or other H_1_R antagonists for melanoma (Fritz et al. 2020a).

In a study with rats, loratadine did not show a significant effect on the skeletal system at lower doses (Folwarczna et al. 2019). Lu et al. (2021) used desloratadine as an antagonist to the 5HT_2A_ receptor in mice which might have potential for Alzheimer’s treatment. In another trial with mice, loratadine was shown to inhibit staphylococcus aureus biofilm formation (Zheng et al. 2022).

Similarly, there were trials with cetirizine on the influence on the bone. Cetirizine decreased tooth movement and decreased osteoclast volume density during orthodontic tooth movement in rats, therefore influencing tooth movement by inhibiting bone resorption (Meh et al. 2011). A study on bone remodeling after calvarial suture expansion in rats came to similar conclusions: by inhibiting osteoclast activity, cetirizine facilitated bone formation (Hwang et al. 2020).

Another study was able to test that cetirizine showed antimicrobial activity against 51 strains of bacteria in vitro. In vivo experiments on mice showed potential of cetirizine against *Salmonella typhimurium* (Maji et al. 2017).

Cetirizine may also have a beneficial effect on viral myocarditis by decreasing inflammation and fibrosis in mice (Matsumori et al. 2010).

Fexofenadine was shown to have a positive impact in tumor necrosis factor-α mediated intervertebral disc degeneration and, therefore, also may be an option for other inflammatory-related diseases (Liu et al. 2021).

## Limitations

Due to the way clinicaltrials.gov works as a platform, we needed to rely on the information provided by the study authors. Even though one expects high standards for the documentation and information regarding clinical studies provided on the platform, especially given the strict regulations and legislation described in an earlier section, there is no easy way to verify the data provided by the authors. Data may be incorrect, incomplete, or not up to date. Especially, the latter is a frustrating problem because it makes working with the dataset harder than it should be: For example, in a lot of cases, we were able to match publications to studies only through PubMed and Google search which would not have been necessary if study authors would have updated the information on clinicaltrials.gov after publication. Regarding the actuality of data, the opposite comes with challenges as well: Our dataset is *not* retrospective and definite but rather undergoes changes on a regular basis when study authors update information about their trials which in itself is positive and desired to mitigate wrong and old data. However, because of this phenomenon, an analysis of clinicaltrials.gov is always somewhat outdated as soon as studies are updated and only represents a snapshot of the landscape of trials the way it was when data was downloaded. To gain a better understanding of the updates on clinicaltrials.gov, we created another script (“data/checkForUpdates.ts”) which scans for new and updated studies. From the start of January 2023 to the start of August 2023, 32 studies were updated, and 12 new entries were found that match the previously described search expression.

Even with correct and up-to-date information provided on clinicaltrials.gov, we still faced the challenge of trials not having published results (yet) and the reference to similar publications during literature research may have been sufficient to gain understanding about potential indications and background but is not optimal. Ideally, we would have liked to refer to a corresponding publication for each trial and use case.

As we have also seen, the strategy to use clinicaltrials.gov as a starting point for our analysis and data acquisition is a limitation in itself. While we may have found use cases that have been tested and validated to the point that trials are being conducted on humans, some interesting or more theoretical indications and revelations were only found during literature research (refer to section “Non clinicaltrials.gov related topics”). It is evident that while clinicaltrials.gov may provide a solid starting point, its exclusive use does not suffice to gain an all-comprehensive overview regarding repurposing especially.

Also, the manual annotation of the data comes with its own challenges as well. While in some cases, the use case of the H_1_R antagonists is evident, in other cases, it is less clear and the assignment is more subjective. Similarly, as already described in the Methods section, in some cases, it was not possible to find a clear focus on *one* of the H_1_R antagonists. While we documented the use of multiple drugs, the analysis was based only on the one first mentioned. Furthermore, the categorization into “repurpose” and “classic indication” was difficult for some studies as well. When in doubt, we mostly categorized it as repurpose.

Lastly, even though we reviewed the literature thoroughly, we still cannot guarantee that we included all relevant topics as we mainly focused on clinicaltrials.gov and PubMed as databases. Additionally, we limited ourselves to publications in English and German. Here, the approach to use clinicaltrials.gov may be beneficial as, due to its international nature, we included studies from all around the world, and every study included in the dataset could be analyzed because all data is provided in the English language by clinicaltrials.gov.

## Future studies on repurposing of H_1_R antagonists

H_1_R antagonists like (levo)cetirizine and (des)loratadine seem to be viable candidates for drug repurposing in some areas. All these findings had in common that H_1_R antagonists have a very good safety profile and thus very little adverse effects. Therefore, it is easy and safe to test these drugs for new use cases. Additionally, they are very cheap: the cost of defined daily dose (= DDD = “The assumed average maintenance dose per day for a drug used for its main indication in adults” (https://www.who.int/tools/atc-ddd-toolkit/about-ddd, last accessed September 28, 2023)) in Germany varies between 0,10€ and 0,81€ before taxes in 2021 (Ludwig et al. 2022, p. 712).

With asthma, the effect of H_1_R antagonists only seems to be secondary by alleviating symptoms of allergy and consecutively improving asthma symptoms, especially in allergic subtypes (Yamauchi and Ogasawara 2019). Thus, according to our findings and general consensus, the use of H_1_R antagonists in the primary and only treatment for asthma does *not* seem to be a promising field for research regarding drug repurposing (Church 2017).

In the case of infusion-associated reactions, the literature suggests cetirizine to be a viable substitute for diphenhydramine with the advantages of less adverse reactions and less drowsiness (Durham et al. 2019; Holmes et al. 2021; Beaucage-Charron et al. 2022). Although this is not surprising, as both diphenhydramine and cetirizine are H_1_R antagonists, the use of the newer generation antagonist cetirizine seems to be a good alternative and replacement for the future.

The use of loratadine to mitigate G-CSF-related bone pain is not as definite. While we could find case reports of complete pain elimination (Romeo et al. 2015) and positive results with a combination of famotidine and loratadine (Gavioli and Abrams 2017), other studies found no statistical differences (Moukharskaya et al. 2016; Kirshner et al. 2018). However, we are inclined to agree with Kirshner et al. (2018) that because of its safety and ease of administration, the addition of loratadine could be considered in cases of bone pain. We still see the need for more thorough and placebo-controlled trials, though.

Similarly, the use of cetirizine for the treatment of alopecia (AGA) seems promising but warrants more research. While positive effects were documented, minoxidil still showed bigger effects (Hossein Mostafa et al. 2021). Chen et al. (2022) concluded in their systematic review on the use of cetirizine for AGA that it was effective and safe. They advised future clinical trials with bigger sample sizes and other factors like the evaluation of different concentrations. Another focus of research could also be the difference in effectiveness between topical and oral cetirizine.

For COVID-19, the use of H_1_R antagonists was tested but studies and trials have either been terminated (possibly due to the lack of interest now that the pandemic has abated slowly) or publications are a documentation of “experimentation” during the pandemic. Although results and the general consensus for the use of antihistamines in the treatment of COVID-19 seem to be positive (Hogan Ii et al. 2020; Al-Kuraishy et al. 2021; May and Gallivan 2022), placebo-controlled scientific evidence is difficult to find. Given that the peak of the COVID-19 pandemic is in the past, the use of H_1_R antagonists as a case for drug repurposing will most likely remain a somewhat successful experiment during a time of crisis, but at the current time, we are inclined to argue that further trials may be warranted but difficult to execute. The fact that most studies regarding COVID-19 and H_1_R antagonists have been withdrawn reinforces this point.

Although there is only one trial regarding the use of cetirizine to prevent neuromyelitis optica episodes (Katz Sand et al. 2018), this does seem like a field that warrants future possibilities and should be followed up in larger placebo-controlled clinical trials.

Similarly, the effects of (des)loratadine on breast cancer and melanoma should be correlated and researched clinically after the findings by Fritz et al. in Sweden (2020a, b).

## Implications of this study for future analysis of drug repurposing in the broader perspective

The clincialtrials.gov API provided us with a straightforward and structured way to access study data in a predictable manner, and the use of custom software made data processing, validation, and manual annotation more accessible and less error-prone.

Although our method and software were sufficient for our goals and research points of interest, it may need adjustment for different research questions and is not a one-fits-all solution but rather an example of using such tools.

The time it took for the initial development may not have been faster than a more conventional approach, but the usage of the now-created tools would yield a much faster and more efficient download, processing, verification, annotation, and analysis because necessary steps are encapsulated into the software for automation.

Also, while our method may require more technical knowledge and programming abilities to set up, the use of a custom-created web application with a specially tailored interface is even more accessible and easier to use than data annotation and verification in, for example, Excel.

Currently, the biggest time factor is the manual verification and annotation of trials. While the former is necessary for every analysis and cannot be fully automated, in the future, our method of analysis could be augmented even further by, for example, incorporating artificial intelligence to reduce the need for manual data annotation. One possibility could be the automatic assignment of a use case with regard to drugs of interest as we described in our Methods section by a large language model (LLM), like GPT-4 by OpenAI (https://openai.com/gpt-4, last accessed July 20, 2023). Results of automatic assignment would still need to be checked and verified, but such integrations seem to be the next logical step in automation.

While our method may be beneficial for reducing human errors during editing and annotation of data through purpose-build user interfaces and automation, software bugs, which are flaws in the code of the software and may lead to unexpected behavior, still need to be considered as a possible limitation.

The use of PostgreSQL as a relational database in the context of our novel methodology in combination with our other tools proved to be beneficial, but there are also challenges that may need to be considered for altered use cases. In comparison to an Excel spreadsheet, a database is (in most cases, exceptions like SQLite exist) not a single file that can be transferred easily via email, for example, but rather more of a program itself that needs to be installed. However, it is wrong to assume that a database and an Excel sheet should be used in the same way and a central database does provide many advantages as well. In a scenario where collaboration is needed and multiple people work with the same dataset, a central database could provide the only source of truth for the data and could be hosted on a computer in a network or in the cloud. All the people working on the project could access the same data simultaneously and edit and annotate using the web application, eliminating multiple versions of the same file distributed over many computers.

Because the database is the only place where data is stored, backups of the data are straightforward as well. In our case, we executed a so-called SQL dump (create-backup.ts), which writes all the data contained in the database to a single file and also includes the instructions to recreate the whole database structure if necessary. Furthermore, one could even leverage version control (e.g., “git”) to keep track of changes to the code files and backup files.

It may also be useful to include other APIs into the software for improved ways of data access but at the cost of needing to adapt the software. There is, for example, an API for PubMed which could be used to search for publications or similar literature when incorporated into the web application. Also, with the transition to a newly refreshed website in June 2023 (https://clinicaltrials.gov/about-site/new, last accessed July 20, 2023), clinicaltrials.gov introduced a new API version that, at the time of writing, is still in a beta phase but will be the replacement for the old API that we used. However, clinicaltrials.gov states that “The classic […] API will remain available for some time.” (https://clinicaltrials.gov/data-about-studies/learn-about-api, last accessed July 20, 2023).

Additionally, the script that checks for updates (mentioned in the “Limitations” section) could be expanded further so that it is able to update and augment the database entries accordingly. For the time being, we, however, decided against such functionality because we preferred to work with one single version of the data that did not change and reflected the state of trials at a specific date and time.

Lastly, it is again important to mention that most of the tools used to create our software can be used free of charge because of open-source licenses.

**Fig. 13:**
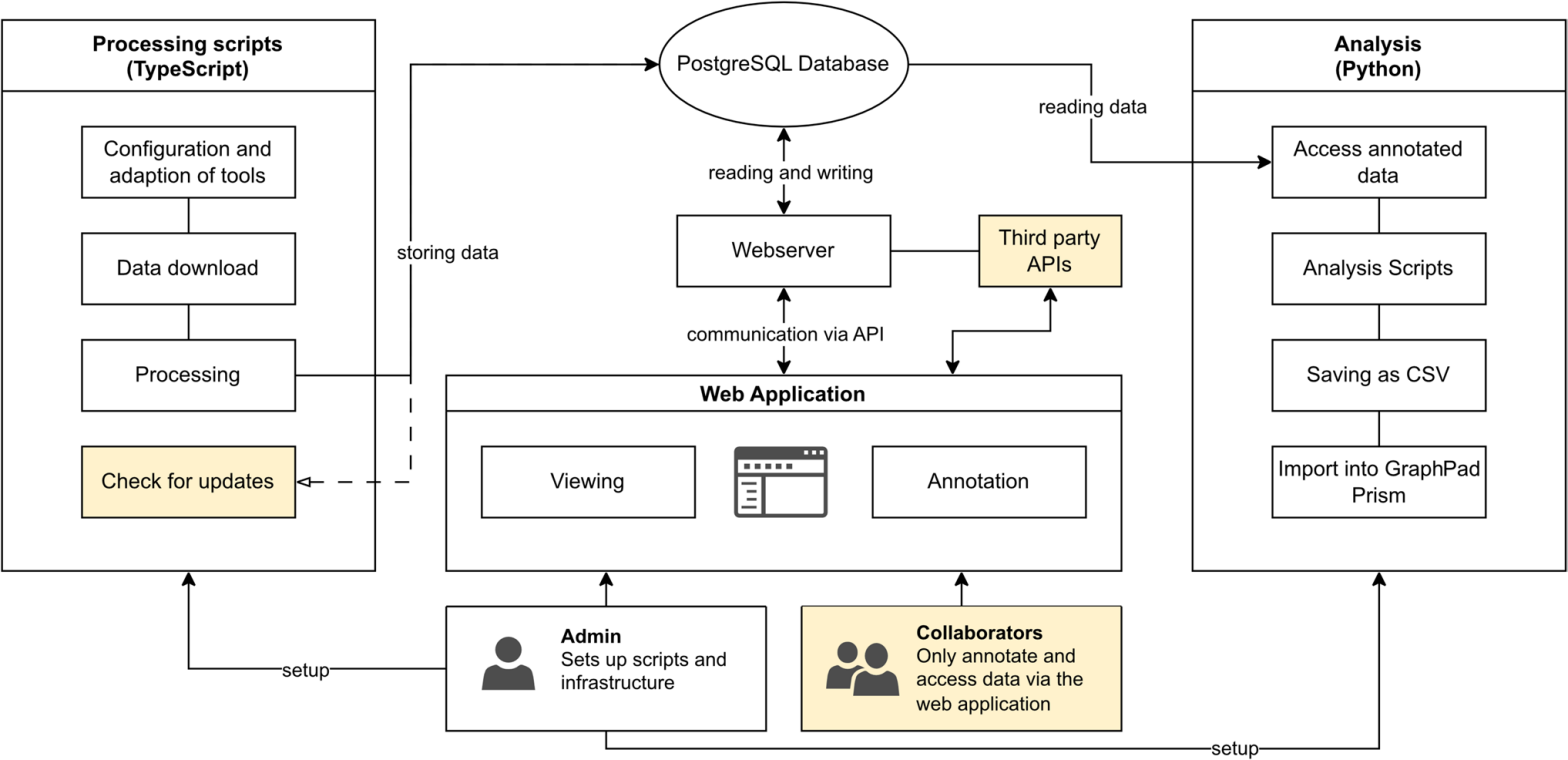
This diagram shows an overview of our software stack and potential enhancements, which are marked in yellow. Refer to the full text for more detail

Figure 13 summarizes the components of our software and highlights the discussed but not yet implemented enhancements in yellow.

To conclude, we would like to highlight the following take-home messages:

- Repurposing of well-known drugs like H_1_R antagonists is an important strategy for the future of pharmacology.
- With our custom-coded software, we were able to identify relevant repurposing indications (including, for example, G-CSF-related bone pain, AGA, or IARs) on clinicaltrials.gov; however, only a small number of studies had published results. More potential areas were elucidated during literature review.
- Our software can be used to support similar research on drug repurposing in the future.

### Supplementary Information

Below is the link to the electronic supplementary material.

Supplementary file1 (DOCX 253 KB)

## Data Availability

All source data for this study are available upon reasonable request.

## Abbreviations

AGA: Androgenetic alopecia
API: Application programming interface
CNS: Central nervous system
DDD: Defined daily dose
G-CSF: Granulocyte colony-stimulating factor
H_1_R: H_1_ receptor
JSON: JavaScript Object Notation
ORM: Object-relational mapping
