# Supplementary material for: Repurposing of H1-receptor antagonists (levo)cetirizine, (des)loratadine, and fexofenadine as a case study for systematic analysis of trials on clinicaltrials.gov using semi-automated processes with custom-coded software
Source: Naunyn Schmiedebergs Arch Pharmacol. 2023 Oct 23;397(5):2995–3018. doi: 10.1007/s00210-023-02796-9 (PMC11074024; doi:10.1007/s00210-023-02796-9)
Supplement: Supplementary file 1 — Supplementary file1 (DOCX 253 KB) [file 210_2023_2796_MOESM1_ESM.docx]

# Supplemental data

# Repurposing of H_1_-receptor antagonists (levo)cetirizine, (des)loratadine and fexofenadine as a case study for systematic analysis of trials on clinicaltrials.gov using semi-automated processes with custom-coded software

# Tim Specht and Roland Seifert

**Supplemental Figures**

Supplemental Figure 1 This chart shows the absolute number of studies grouped by year based on the date it was first posted on clincaltrials.gov, color coded by drug name. Also refer to Figure 5 for the same graph based on study start date.

Supplemental Figure 2 This chart shows the distribution of sex eligible for participation in a study grouped by the drug name. For each drug, the distribution was calculated individually as a ratio between 0 and 1.
